# Assembly and Interconnection Technologies for 3D Plastic Circuit Carriers: An Overview of Technologies, Materials, and Applications

**DOI:** 10.3390/mi16090980

**Published:** 2025-08-26

**Authors:** Kai Werum, Wolfgang Eberhardt, Dieter Reenaers, Thomas Mager, Mika Endl, André Zimmermann, Wim Deferme

**Affiliations:** 1 Hahn-Schickard-Gesellschaft für Angewandte Forschung e.V., 70569 Stuttgart, Germany; wolfgang.eberhardt@hahn-schickard.de (W.E.); mika.endl@hahn-schickard.de (M.E.); 2 Institute for Micro Integration (IFM), University of Stuttgart, 70569 Stuttgart, Germany; andre.zimmermann@ifm.uni-stuttgart.de; 3 Institute for Materials Research, Hasselt University, Martelarenlaan 42, 3500 Hasselt, Belgiumwim.deferme@uhasselt.be (W.D.); 4 Division IMOMEC, IMEC vzw, Wetenschapspark 1, 3590 Diepenbeek, Belgium; 5 Fraunhofer Institute for Mechatronic Systems (IEM), Zukunftsmeile 1, 33102 Paderborn, Germany

**Keywords:** assembly, 3D, interconnection technologies, packaging, printing, mechatronic integrated devices (MIDs), laser direct structuring (LDS)

## Abstract

This paper aims to present an overview of the state-of-the-art materials and technologies that can be used to create electronic circuits on 3D plastic carriers also known as 3D electronics. Strategies for print-based and laser-based 3D electronics will be discussed as well as the techniques to apply the circuit carrier and the way interconnection technology can be used to combine electronic components on top of the circuit carrier. A basic explanation of the functional principles, materials, and applications is given for different substrate and interconnection technologies. The aim is to make it easier to compare different technologies and its required materials to make the right decisions on what technology is best suited for the job. For this purpose, comparison tables for 3D plastic circuit carrier technologies and substrate materials considering their temperature stability were created. It can be concluded that there are a lot of influencing factors that determine which technologies are best suited for application. The most important factors are the 3D complexity and the field of application, the required structure size of the circuit, and the required production quantity.

## 1. Introduction

The field of smart devices is growing rapidly [1]. Every product has the potential to become smart by means of integrated electronics. The creation of new, creative, and intelligent products is enabled by cost reduction, miniaturization, novel materials, and integrated sensors with data processing. The latter requires a printed circuit board (PCB). However, using conventional techniques that can only be realized in two or two and a half dimensional forms is limiting design and integration options.

New electronics integration techniques, such as printed electronics (PE), structural electronics (SE), and 3D electronics have been widely investigated in recent years. Where PE is using printing techniques to deposit conductive traces only, SE is combining PE with assembly of surface mount device (SMD) components. Three-dimensional electronics in the end tries to combine the previous ones on (or in) a 3D structure. These technologies overcome the main disadvantage of traditional PCBs with regard to form factor. By adding a third dimension to digital circuit design, integration density can be improved and new concepts, like the integration of electronics or sensor and lighting components in specific orientation directly in the housing of a product, can be realized. Similarly, mechanical or fluidic functions can be integrated directly. The market for 3D plastic electronics is broad, with a variety of application domains such as medical technology, automotive, aerospace, industrial technology, and communication and consumer electronics as potential target markets. Since some technologies have been on the market for several years, 3D design tools have become available [2,3,4,5,6]. However, with the multitude of applications, the requirements vary as greatly as does the technical performance of the different technologies available. As a result, it is a challenge to determine the best manufacturing technique for each application.

**Figure 1:**
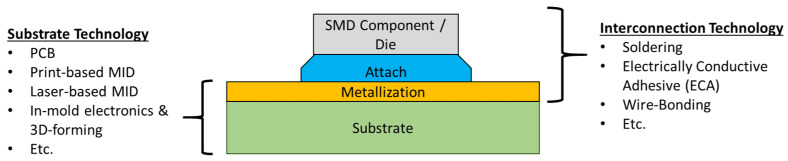
Overview of the proposed terminology for assembly and interconnection technology for 3D plastic circuit carriers to create 3D electronics. Substrate technology is focusing on the deposition of conductive traces and circuits on the plastic carrier, whereas interconnection technology is linked to the attachment of SMD components onto these circuits.

This review paper gives an overview of substrate and interconnection technologies used to manufacture plastic-based 3D electronics and compares their advantages and disadvantages. The technologies are divided into distinct classes based on their application mechanism. For each technology, the working principle is explained, best-suited materials discussed, and conventional and possible applications presented. In Figure 1, the terminology is provided to clarify what is meant with different parts of the process.

First, the focus is on print-based 3D electronics. Within this domain, application methods to create conductive traces are distinguished into contact printing and non-contact printing. Non-contact printing techniques such as inkjet printing, valve jet printing, and aerosol jet printing (AJP) will be discussed. These methods can be used to print directly onto a 3D object and are also referred to as digital printing.

Contact printing on the other hand is referred to methods where tools, stencils, screens, or masks are involved, which lead to physical contact with the substrate. They are mainly used to create conductive traces on flat substrates. Although the conductive traces are formed on initially flat substrates, they are relevant, because by means of techniques such as in-mold electronics and 3D forming, it is possible to transform the 2D circuits into 3D electronics.

Secondly, laser-based 3D electronics using laser-based MID technology is discussed. MIDs (mechatronic integrated devices or molded interconnect devices) are three-dimensional rigid circuit carriers. At the beginning of MID development, the basic 3D body was manufactured primarily using injection molding. Nowadays, the term mechatronic integrated devices is used in a broader sense, since the 3D bodies are not only manufactured by injection molding of thermoplastics exclusively anymore but also with other materials, e.g., ceramics [7,8] or thermosets [9,10], as well as different forming processes like additive manufacturing. Generally, the process sequence for MIDs starts with building a 3D-shaped basic body followed by creating 3D conductive tracks and assembly of electrical components like, e.g., SMD.

With laser direct structuring (LDS), 3D conductive tracks are created using a laser beam for activation of 3D surfaces followed by electroless plating. The 3D-shaped basic body is mainly fabricated by injection molding but also additive manufacturing, and subsequent coating with a LDS lacquer or using a new 3D printable LDS resin can be utilized [11]. Other laser-based technologies like semi-additive processes as well as selective surface activation processes are also known for generating 3D conductive tracks.

Furthermore, alternative structuring processes like two-shot MID technology, hot embossing, and plasma-based technologies are briefly discussed.

Lastly, technologies to assemble and connect electronic components onto 3D electronic devices are discussed. Nowadays, a lot of smart products can be made using flexible substrate materials. However, most conventional electronic components are still only available in a rigid form factor, mainly as SMD or bare dies. Therefore, interconnection technologies such as conductive gluing, soldering, and wire bonding are discussed with regard to the substrate technologies.

After all the technologies and techniques are discussed, common and innovative applications for each are presented in a broader context. At the end of this review paper, a summary table is provided presenting the main differences, potentials, and similarities of each technology. This allows users of the technology to easily and quickly identify the best technology for their application.

## 2. Print-Based 3D Electronics

The domain of printing technologies can be divided into non-contact and contact printing techniques [12]. In contact printing techniques, pre-formed patterned structures with inked surfaces are brought into physical contact with the substrate. In non-contact printing techniques, the printing material is dispensed through a nozzle, and patterns can be created by moving the nozzle across the substrate. Non-contact printing techniques have become more attractive over the years thanks to their simplicity, lower cost, speed, adaptability to the manufacturing process, reduced material waste, high resolution patterns, and ease of control by adjusting various print parameters [12].

To create printed 3D electronics, a two-step process is required. Depending on the applied printing technology (contact vs. non-contact), these two steps alter. When contact printing techniques such as screen printing are applied, the first step is to print the interconnections onto a flat substrate, typically flexible or stretchable to allow the forming in step 2. This second step can be performed with in-mold electronics or 3D forming. When non-contact printing techniques such as inkjet printing are applied, first the object is formed, and only after that, the second step requires the deposition of the printed circuit layer onto the 3D object. The reason to swap the steps as compared to contact printing is mainly related to the stretchability of the conductive circuit materials applied onto the substrates. For non-contact printing, such as screen printing, ink formulations are used that are stretchable after deposition, whereas for the typical non-contact printing, due to the use of small nozzle openings, typically nanoparticle-based inks are applied, and after sintering—to make the circuit conductive—these conductive circuits are less stretchable and are prone to breakage upon 3D forming. On the other hand, having a nozzle-based, non-contact technology, depositing the ink formulation on a 3D object is possible, which is less the case with the contact printing technologies.

The right selection of the interconnection technologies is important in 3D electronics, because they determine the durability of the newly created device. A bad material choice or a bad application technique can cause failure of the device during the fabrication process or while in use by a consumer. Various printing technologies require different printing parameters, such as viscosity, surface tension, conductivity and compatibility with the solvents. These parameters, together with the printing methodology and adequate materials, will be discussed in the next sections.

### 2.1. In-Mold Electronics by Means of 3D Forming

#### 2.1.1. Introduction

In-mold electronics (IME) manufacturing is a combination of plastic molding and functional materials printing. It adds the ability to obtain electronic functions on plastic formed shapes. Thus, features like sensors, controls for illumination, and communication can be placed on these surfaces. This method can lead to a 70% reduction in weight and up to 90% reduction in thickness compared to conventional techniques [13,14].

The first part of the manufacturing process is printing (Section 2.1.2). Here, the conductors and contact paths are printed onto the polymer film on a 2D surface. The big advantage here is that 2D printing techniques are widely available and are highly industrialized. The next step in the manufacturing process is surface mounting, which will be described in Section 5 in this review paper. Component bonding in IME relies on conductive adhesives instead of solder paste. The biggest advantage is that the curing temperature can normally remain lower than 150 °C (see Section 5).

Now the object can be formed (Section 2.1.3). This is carried out by vacuum or thermoforming. The maximum temperature depends on the polymer film. This temperature is typically below 150 °C. The maximum pressure is typically below 8 MPa. The biggest problem with this step is deformation and strain for the components and the printed paths. Here, cracks can be easily formed that lead to failure.

The final step is injection molding. Here, all the components are subject to elevated temperatures and pressures. In this process step, the material compatibility of polymer film and injection molded thermoplastic has to be considered. As the molded IME structure cools, a thermal expansion mismatch can lead to stresses in the components and their connections [13,15].

#### 2.1.2. Contact Printing by Means of Screen Printing

Contact printing typically is applied on flat substrates. After that, typically 3D forming is performed to achieve the conductive circuit on a 3D shape. There are several contact printing techniques that could be used. However, in this section, we only discuss screen printing.

Screen printing is by far the most popular contact printing technique for planar substrates used in the electronics industry, because it has been widely researched and used in recent years and achieves very high throughput [12]. In screen printing, a squeegee and blade are used to spread a paste over a screen and transfer the paste onto the substrate. The screen is permeable in some areas and impermeable in others. This makes it possible to print distinct designs on the substrate. By adjusting the thickness of the mesh, which is commonly made of stainless steel or polyester, or by tuning the emulsion layer, the final thickness of the design can be changed [16].

**Figure 2:**
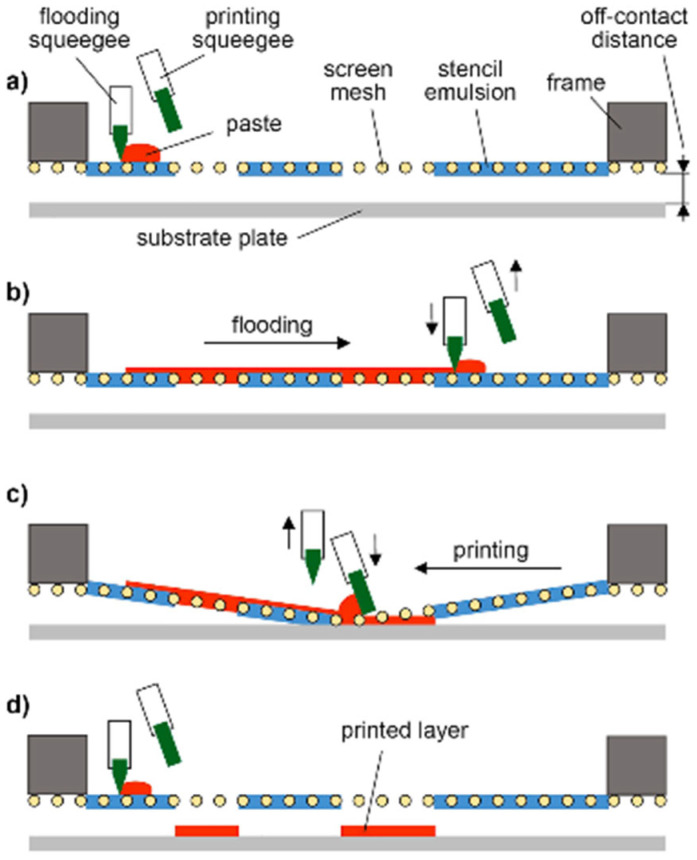
The flatbed screen printing process in different steps. (**a**) Starting state with paste applied to the screen, (**b**) flooding step to distribute the paste across the screen, (**c**) printing step in which the paste is applied to the substrate using a squeegee, (**d**) final state with the printed layer [17].

Figure 2 shows the flatbed screen printing process in four steps. This process can be repeated to print multiple layers. Flatbed screen printing is mostly used in small laboratories. Higher speeds can be achieved by using a roll-to-roll (R2R) fabrication process. However, these screens are very expensive and difficult to clean [12].

Screen printing can be used with various printing materials and substrates. Commonly used substrates for screen printing are metals, wood, fabric, glasses, ceramics, paper, and various polymers. However, to enable flexible electronics, flexible substrates are needed.

There are three types of substrates for flexible electronic devices—thin glass, metal foils, and plastics [12]. Thin glass is bendable, but the intrinsic brittle property limits its utility in flexible electronics. Metal foils can sustain high temperatures and enable the use of inorganic materials to be deposited on it, but its high cost and surface roughness are not ideal for the manufacturing of flexible electronics. Plastic materials are highly bendable and potentially stretchable, transparent, and emissive, which makes them ideal for flexible electronics. Plastic materials offer a good balance of physical, chemical, mechanical, and optical properties. In addition, it enables the large-scale and low-cost production of flexible electronics (e.g., R2R manufacturing). However, the use of plastic substrates is limited by the lower heat shape resistance temperatures and glass transition temperatures (Tg). It should be noted that the choice of substrate is also influenced by the application itself such as RFID, sensors, OLEDs, etc. In Table 1, there are a few polymer types listed with their respective molding temperatures.

The most used polymer types are PC (85%) and PET (10%), but other types (5%) like ABS, PC/ABS, PMMA, TPU, PS, PE, PBT, PA6, and PA66 are also used [13,18,19,20].

The inks used for screen printing contain fillers, binders, and solvents in specific proportions to ensure a good quality ink. The binders and solvents can be divided in two categories—organic and aqueous. Organic binders prevent mass production, because these materials are toxic and harmful to the environment. Therefore, research has recently been conducted in the development of “green-inks” using sustainable binders and solvents. However, the main challenge of “green-ink” lies in the matchmaking of binders and substrates. Deionized water is proposed as a promising new solvent for future screen-printing inks. Thanks to the development of “green-inks”, screen printing is becoming even more recommended for large scale production of batteries, supercapacitors, and sensors [21].

The binders and solvents inside the screen-printing ink determine the viscosity, rheology, and functionality. They enhance the adhesion of the ink to the substrate without clogging the mesh of the screen. It should be noted that the substrate choice also has an influence on the viscosity of the ink. Mechanical strength, high impact toughness, superior elasticity, corrosion resistance, tunable viscoelasticity, and outstanding chemical and thermal stability are all important properties of binders [21].

In the case of printed electronics, conductive inks need to be developed while having the same properties as regular inks. Usually, binders cause the electrical conductivity to decrease. Therefore, conductive additives such as carbon black, acetylene black, graphite, and carbon nanotubes are added to improve the electrical conductivity of the ink [21]. An overview of the most commonly used binders, conductive additives, and solvents can be found in [21]. In general, it can be said that silver nanoparticle and microparticle/flake inks are most commonly used. Ag nanowires (AgNW) inks are proposed to replace silver nanoparticle (AgNP) inks in the near future, because it allows conductive network formation at low material loadings, which gives more freedom in the ink rheology. Finally, carbon nanotube inks (CNT) are even more promising because of their excellent elastic and electrical characteristics and cost-effective production. However, strong interparticle attractions make steady dispersion problematic [22].

The essential characteristics needed for IME is the extensibility of the printed structures. Therefore, current conductive inks are composed of silver flakes mixed with elastomeric components. When this type of ink is stretched by 40%, it can lose up to 10 times its conductivity. Another problem can be the high temperatures and deformation in manufacturing because of the used elastomers.

An extra promising material is screen-printable silver molecular ink, because they can produce thin, strong, and conductive traces. Also, more generally, silver and copper molecular inks can be used.

Furthermore, a silver oxalate-based molecular ink can be sintered with steady-state, high-intensity broadband UV light [15] to produce highly conductive silver metal traces. Table 2 lists other types of ink that can be used.

Now, one big problem of IME is the resistance change while molding. So, it seems logical that the current research is still carried out in the field of highly stretchable conductive inks for three-dimensional electronics [24].

Here, eutectic gallium indium alloy (EGaIn) would be a good candidate, because this type of metal is inherently stretchable without the use of polymeric filters. The big disadvantage of using this material is that it is not a compatible ink for printed electronics manufacturing because of its low viscosity, high surface tension, and high tendency to oxidize in air [25].

The advantages of screen printing in comparison to other printing techniques are its low cost, excellent operability, simple manufacturing process, rapid fabrication, high throughput, and large-scale production. The major disadvantage of screen printing are its limited functional ink choices and the limitation to develop new screens inside a standardized production process [21]. However, in the past few years, a lot of research has been conducted into possible materials for screen printing functional inks. Table 3 shows the most important screen-printing specification characteristics and their scale.

The viscosity of the paste and the surface tension of the substrate are essential for complete paste dispensing through the screen mask. Usually, higher viscosity inks are more compatible than lower viscosity inks, because they are more likely to run through the mesh rather than dispensing out of the mesh. However, lower viscosity inks are preferred to print smooth line edges and obtain higher resolution. Moreover, using low-viscosity inks reduces the chances of blocking the mask. Screen printing also enables the printing of thick layers with lower resistivity [12].

Screen printing can be used for many state-of-the art applications such as manufacturing micro-batteries, micro-supercapacitors, and micro-sensors [21]. However, there are still challenges to be solved. For micro-batteries in particular, it is still difficult to print the electrolyte membrane. For micro-supercapacitors, the formulation of the ink still needs to be researched. Micro-sensors can still be improved by adding modified nanomaterials inside the inks to improve detection sensitivity.

**Figure 3:**
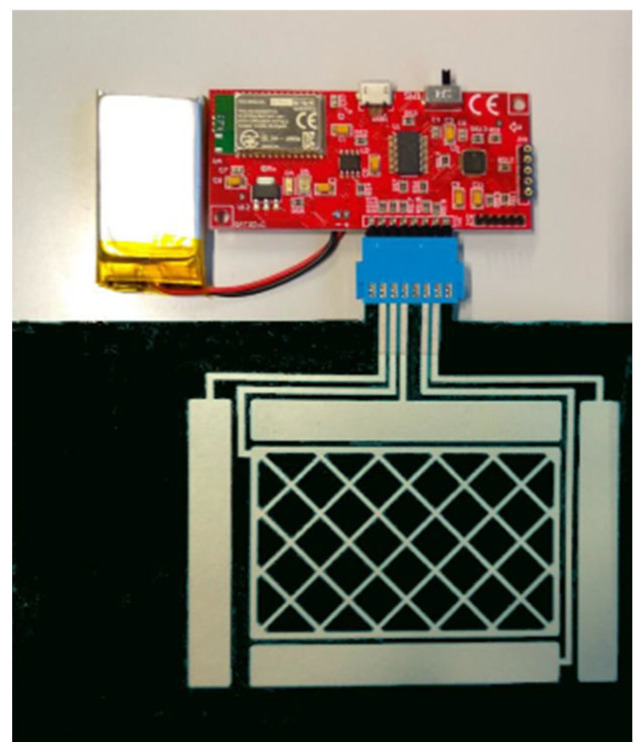
3D gesture recognition sensor system [16].

High conductive flexible electronic circuits have been established for the development of touch, temperature, pressure, and humidity sensors [12]. For example, a wearable textile 3D gesture recognition sensor has been developed [16]. Therefore, a capacitive touchless sensor has been printed with screen printing. This is an example of how screen printing can be used to create integrated applications. Figure 3 shows the sensor used in an integrated gesture recognition system.

Integrating electronics into wearables, such as textiles, is one of the most popular domains of screen-printing electronics. Electroluminescent (EL) lamps on textiles are one example that is mainly being produced with screen printing. The authors of [21] reported that stretchable (electroluminescent) EL lamps can be manufactured with thermoplastic polyurethane directly on textiles thanks to screen printing technology. In [26], an EL lamp has been screen-printed directly on to woven polyester cotton fabric to create a smart fabric lamp. The EL lamp consists of six individually printed layers, and the printed structure has high durability while maintaining the flexible and breathable properties of the fabric.

Other contact printing technologies that could be used to deposit the circuit layer are (among others) flexography, offset printing, and gravure printing. Those technologies achieve similar results in terms of resolution, line width, and line thickness as screen printing but are based upon different technologies to deposit the conductive material; however, this is still in a contact manner.

Following the first step of depositing the circuit layer onto the flexible substrate, a second step, i.e., 3D forming, is needed.

#### 2.1.3. 3D Forming

Three-dimensional forming is a technique in which a one-time deformable foil is stretched around a 3D object. The main use case of 3D forming is making common household items “smart”. For example, by forming a foil that has extra capacitive touch buttons and/or led indicators that can give extra information. Most articles state that they use vacuum forming or thermoforming. They sound like two completely different techniques, but vacuum forming is actually a type of thermoforming, so we will generalize both by stating 3D forming further. The vacuum forming is executed around 100–160 °C for most use cases, but this can vary a lot depending on the type of substrates [27,28]. The sheet/foil is first heated to above its glass transition temperature. During heating, the temperature is measured. When the deformation of the sheet is visible, the sheet is stretched over the mold, and a vacuum is created between the sheet and the mold. This is to ensure that the sheet takes over the shape of the mold. When the sheet is cooled down enough, the vacuum is removed, and the sheet remains in the shape of the mold.

As mentioned above, good candidates for thermoformed printed electronics are PMMA, PP, and PC. These materials have a relative high melting point and heat shape resistance, which is required to maintain their shape after forming in a wide range of environmental conditions. Moreover, these polymers have a low thermal expansion coefficient, resulting in more stable behavior during the forming process where heat is usually applied by IR heaters or hot air [29].

With most techniques for creating 3D electronics, the substrate is rigid after all production steps are completed. However, this is not the case when using styrene-ethylene-butylene-styrene (SEBS) as the substrate. SEBS is a stretchable thermoplastic elastomer. It can be elastically stretched as far as 600% when it is used as the 3D electronic device. It is also suitable for embedding the electronic components inside the substrate [28].

**Figure 4:**
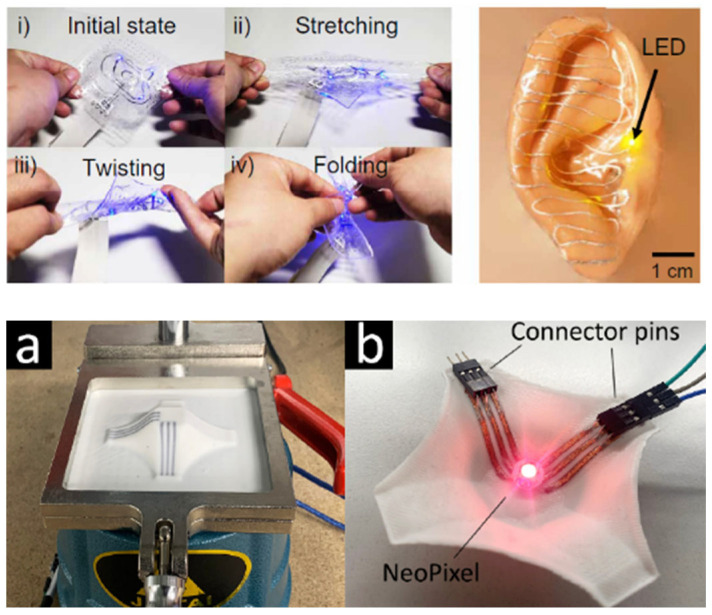
(**Top**): Stretching capabilities (**left**) and application example (**right**) of the device [28]. (**Bottom**): Demonstrator in vacuum forming machine (**a**) and with connected NeoPixel light (**b**) [27].

The left side of Figure 4 gives an overview of the stretching capabilities of the device. On the right side, it is shown that there is still enough rigidity to stay attached to something like an ear. SEBS substrates are specifically interesting for stretchable encapsulated IME, when stretchable inks are printed onto these substrates and later overmolded with a second layer of SEBS [30].

The foil itself can also be 3D printed with FDM printers. This has some advantages over conventional foils. It is possible to vary the thickness of the foil easily and use multiple materials in one foil. The circuit is also completely embedded inside the foil. For example, this makes it possible to print the foil with regular polylactide (PLA) filament and embed the traces of the electronic circuit in the foil by printing them with conductive PLA [27]. This is one of the most recent advancements in 3D vacuum forming.

#### 2.1.4. Applications

Applications of screen-printed IME are currently well known in the automotive industry, including touch panels, electrochromic displays, lighting (such as electroluminescent features), and antennas. In this particular field, IME enables seamless human interfaces with fewer mechanical parts, reduced weight, and increased design freedom. A prominent example are touch buttons integrated into car dashboard and center consoles [31,32,33,34,35].

Beside automotives, IME is expanding within the field of medical devices and robotics. Wearable (on skin) medical devices benefit from IME technology, since they are lightweight, flexible, and ergonomically shaped, increasing the patient comfort during monitoring. Electrocardiogram (ECG), temperature sensing, hydration tracking, or even wound monitoring (e.g., pH sensing) are all possible by means of printed IME. Additionally, IME facilitates the design of disposable or semi-disposable applications, since the technology enables low-cost production and modularity [36,37].

Although the technology is still emerging, there are several standard electronic component packages that are already proven to be compatible with IME. In this way, applications can be developed. The following packages are suited for IME [13,38,39,40]:

- Most SMD packages (e.g., 0603, 0805);
- Quad-Flat-No-Lead (QFN) package;
- Land-Grid-Array (LGA) package;
- Chip-Scale-Package with few contacts.

### 2.2. Non-Contact Printing

As mentioned in the introductory part of this section, non-contact printing could be applied as well to achieve the interconnection technology onto the 3D object. In this case, typically first, the 3D forming is performed, and after this, the non-contact printing is carried out. There are several non-contact printing techniques that could be used. In this section, we discuss three of them in a bit more detail, i.e., valve jetting, inkjet printing, and aerosol jet printing.

#### 2.2.1. Valve Jetting

Using jetting valves creates fast, continuous, and non-contact dispensing of small dots possible, providing a high degree of accuracy and repeatability. The jetting process starts with filling a small chamber with material and ejecting by shooting a tappet through the chamber. To become a very fast tappet movement, a piezo or high energy pneumatic is used. This technique causes the material to come out of a nozzle [41].

A pneumatic valve is used for on–off jetting control to lift the needle by using air pressure. These pneumatic valves have several drawbacks, such as a relatively short lifespan, and their maximum spraying frequency can only be ca. 100 Hz, which is relatively low. For these reasons, the piezo-electric (PZT) powered non-contact dosing method is emerging as a worthy alternative method due to its high efficiency, fast response, and highly accurate operation.

**Figure 5:**
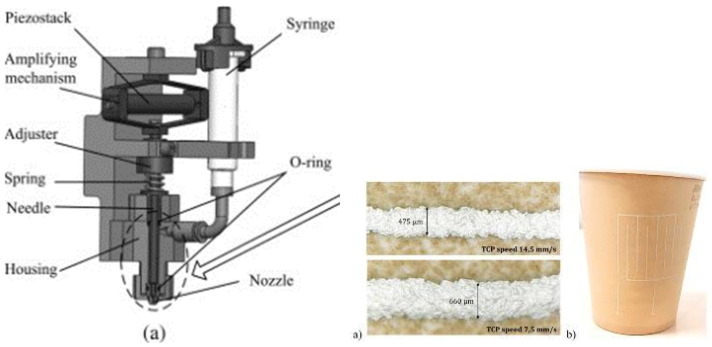
(**left**): Piezo-driven dispenser for valve-jet printing [42]. (**Right**): Printed line width for 50% and with a constant DL of 19 ms [43].

Figure 5 (left) displays a schematic representation of the typical piezoelectric dispenser with needle impact. In the PZT drive dosage system, a piezo stack actuator is attached to a displacement amplifier. This actuator acts as the major drive component. This method makes precisely controlling the amount of liquid material expelled from the orifice much more accurate and efficient.

Due to the ability to scale up for mass production, the valve jet printing method is commonly used for printed electronic applications. Nevertheless, valve jet printing also has several limitations to expand its applications, for example the requirement for low-viscosity printable inks.

Patterns with less residual material after evaporation can be the consequence of a droplet deposition based on low-viscosity inks (<10 mPa·s). By using low-viscosity materials, the chance of uneven widths a thickness increases considerably, depending on the ink evaporation behavior or substrate conditions.

To satisfy the recent application standards, printing methods using high-viscosity inks or a fine printing patterns are needed. These patterns have a width of just a few micrometers. These requirements exceed the capabilities of conventional inkjet printing. To obtain better electronic properties of printed patterns, the required ink viscosity must be very high, more than 1000 mPa·s, to increase the solid content.

By using inks with a higher viscosity characteristic, the printed cartridges are slightly less affected by the surface condition of the substrate. For this reason, high-viscosity ink dispensers have been widely used for valve jetting technologies [42].

Table 4 shows the most important valve jet printing specification characteristics and their scale.

The major advantages of valve jet printing are the high operating speeds up to 3000 Hz with high accuracy, the capability to dispense consistently, even on substrates with an irregular surface, orientation, or an unusual geometry. The limited pressure caused by collision of the needle is a major drawback of valve jet printing.

It is established that the printable viscosity ranges of contact dispensing methods are slightly higher than the jet dispensing methods [42].

When the modern available direct writing technologies are compared with each other, it is noticeable that the conventional inkjet and paste piezo jetting methods allow a high precision for dispensing a broad range of available inks. Some examples of those inks are photoelectric, conductive, or dielectric inks for various electronic applications.

For high-precision printing applications on 3D substrates using unit drop spreading, a multi-axis robot coupled with a piezo-jetting printhead and a semi empirical model is commonly used. This empirical model is capable of predicting the line width as a function of the printing velocity in combination with the diameter of unit drops and the frequency of the ink jetting. Direct correlations between printed lines, printing parameters, and their electric resistance can be established by combining all of the data. Integrating the model in the multi-axis robot control software ensures a correct ink jetting frequency as a function of a target line width. The authors of [43] show the first printing tests on a 3D paper cup. This test concludes that the robotic cell in combination with the control software allow printing complete circuits with constant line width and electric resistance.

#### 2.2.2. Inkjet Printing

**Figure 6:**
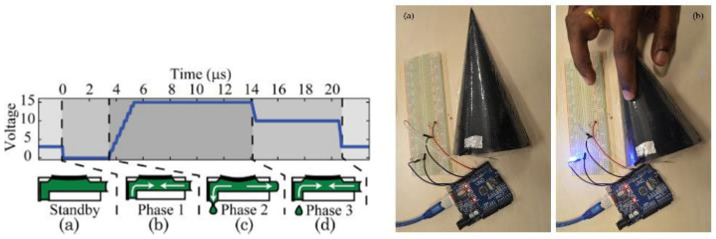
(**Left**): Voltage waveform for inkjet printing [45]. (**Right**): Capacitive touch sensor using inkjet printing and 3D forming [29].

Inkjet printing is the process of jetting controlled, small droplets of ink onto a substrate, without using a mask [44]. The most common inkjet technology in the industry for printing functional inks is drop-on-demand (DoD) piezo-electric inkjet printing [44]. A drop-on-demand inkjet printer works by applying a voltage waveform to a piezo-electric transducer, as can be seen in Figure 6.

Applying a high voltage will push the piezoelectric transducer inwards, which results in an ink droplet coming out of the nozzle because of the higher pressure on the fluid. When the voltage is removed from the piezoelectric transducer, the ink droplet falls onto the substrate [46]. The falling process is called jetting and consists of a rise time in which the fluid expands, a dwell time, and a fall time in which the fluid contracts. Jetting behavior should be controlled precisely to avoid satellites and the emission of a random spray.

In order to print conductive traces, the droplets that form the printed line should be uniformly distributed. In advanced printers, the printhead can be customized to fit more nozzles, control the drop volume and viscosity of the ink, and use different types of inks. Other printing parameters that can be changed are the drop spacing, jetting frequency, printing speed, printing height (typically 1 mm), cartridge temperature, substrate temperature, and the number of layers. The behavior of the ink during jetting depends on the Reynolds number (Re) and the Weber (We) number [46].

Printable conductive materials for inkjet printing can be divided into inorganic and organic materials. Most of the printable materials are inside a solvent, therefore having specific rheological properties to allow proper printing on a variety of substrates.

The first type of ink that can be used for inkjet printing are nanoparticle inks. Nanoparticle inks are wet inks, typically consisting of 90 *v*/*v*% solvent and 10 *v*/*v*% functional material. The solvent is designed to correspond with the correct rheological properties, commonly defined based on the Ohnesorge number, which relates to the jet ability and spray ability of fluids. The nanoparticles are generally protected from agglomerating by a capping agent, which is applied as a thin film around the individual particles. This capping agent is made of a polymer (usually PVP, PEG, or similar), and the melting point of this polymer defines the minimum sintering temperature. When sintering is conducted, two main phenomena are happening. Solvent removal reduces the wet layer thickness after depositing, bringing the nanoparticles together to form a compact dense nanoparticle layer. At the same time, the capping agent starts to melt, so the individual nanoparticles start to touch each other, initiating Ostwald ripening and aggregative particle growth, forming conductive pathways [47]. This process is enabled through various sintering methods such as thermal, laser, flash, microwave, electrical, or chemical sintering [46]. A disadvantage of the sintering process is that when using conventional sintering methods, high temperatures (up to 60 min at 100–200 °C) are needed to remove the solvent. Newer techniques like flash sintering eliminates this limitation, opening opportunities towards temperature sensitive substates, but at a cost. To obtain conductive traces, metals with low resistivity such as silver (Ag), copper (Cu), and gold (Au) are preferred. Nowadays, Ag-inks are the most popular thanks to their good physical and electrical performance on plastic substrates, low oxidation rate, and high conductivity. Copper is less popular due to its oxidation rate after printing [48].

The second type of ink that is suited for inkjet printing is MOD (metal–organic decomposition) ink [49]. This ink consists of highly concentrated metal salts dissolved in solvents. After the salt is printed onto the substrate, heating is applied, and this converts the salts into conductive metal tracks [48]. It should be noted that the amount of heat required is less than the sintering process needed with the nanoparticle-based inks.

The third type of inks are organic polymer inks that use materials such as polyacetylene, polypyrrole, polyphenylene, polythiophene, polyaniline, polyaniline doped with camphorsulfonic acid, and PEDOT:PSS (3, 4-polyethylenedioxythiopene-polystyrene sulfonic acid) [12]. These organic materials are doped with p and n type material to become intrinsic conducting polymers. The chemical structure of these inks should be adapted to obtain the desired mechanical and electrical properties. In particular, PEDOT:PSS has a high electrical conductivity of 300 S/cm (Siemens per centimeter) [12]. However, in comparison to conventional metals such as Ag, which has a conductivity of 6.30 × 10^7^ S/m (Siemens per meter), PEDOT:PSS still exhibits significantly lower performance [12]. Nonetheless, the incorporation of silver nanowires into PEDOT:PSS inks can significantly increase their electrical performance. PEDOT:PSS has been widely investigated, because it can replace the most used transparent conductive electrode called ITO (indium tin oxide). ITO is expensive, hard, and fragile, and it requires high temperatures to produce, which is not suited for 3D electronics fabrication [29].

The substrates used for inkjet printing are the same as those of screen printing. However, it should be noted that the sintering process requires high temperatures, and therefore, some thermoplastics may not be suited as a substrate.

Table 5 shows the most important inkjet printing specification characteristics and their scale.

The advantages of inkjet printing are its low operating cost, good resolution, and high precision. Additionally, inkjet printing has no material wastage, can print on large areas, and no controlled environment is required. It is a single step process in which no mask is required in order to apply the conductive patterns. Disadvantages are the limited substrate and ink choices, because the ink should have a low concentration of binders with a high viscosity in order to prevent the blocking of the nozzle [12,21,44].

Inkjet printing can be used for various applications, such as the fabrication of touch sensors, light-emitting diodes (LEDs), OLEDs, solar cells, organic thin-film transistors (TFT), RFID tags, etc.

A first application, demonstrated in [50], produced highly conductive LED circuits on photo paper and PET using inkjet printing with Ag nanoparticles sintered at low temperatures (<100 °C).

Secondly, ref. [29] has combined PEDOT:PSS ink with additional solvents to achieve the desired inkjet printing properties for printing conductive traces that are one-time stretchable up to 40% [29]. Three different loadings of the PEDOT:PSS ink were tested, as follows: 10%, 20%, and 30%. It was found that all inks were printable without blocking the nozzle, and a linewidth of 44 µm and a drop spacing of 40 µm were achieved. The ink loaded with 10% PEDOT:PSS was the most transparent and could be printed most easily. The ink loaded with 30% PEDOT:PSS was able to reach a sheet resistance of 45 Ohm/sq when printing five layers on top of each other. To demonstrate these findings, a capacitive touch sensor was created on a stretchable substrate, as can be seen in [29]. The achieved sheet resistance after 40% of strain was still high enough (in the range of kΩ) to sense touch input [29].

It should be noted that polymers such as PEDOT:PSS start to degrade while exposed to solar radiation. Therefore, it is important to research the lifespan of the developed ink. It was found that inks with a high amount of polymer degrade more than inks with a low amount of polymer [29].

Other literature has created cost-efficient, ITO-free polymer solar cells based on PEDOT:PSS–Ag (15 nm)–PEDOT:PSS layers (PAP). As mentioned earlier, ITO is not suitable for flexible electronics. Therefore, PAP enables the development of flexible solar cells using PAP multilayers as a transparent electrode [15].

Alternatively, inkjet-printed ITO free OLEDs have been developed by [51]. They used a particle-free Ag ink and used plasma sintering afterwards to create conductive silver traces. The applied method used low temperatures and resulted in good optical transmittance, electrical conductivity, luminance, and flexibility for the developed OLEDs in comparison to ITO-based OLEDs.

#### 2.2.3. Aerosol Jet Printing

Aerosol jet printing (AJP) is a multiuse technique suitable for large-area and fine-feature patterning with the capability using both rigid and flexible substrates with a variety of usable inks [52].

**Figure 7:**
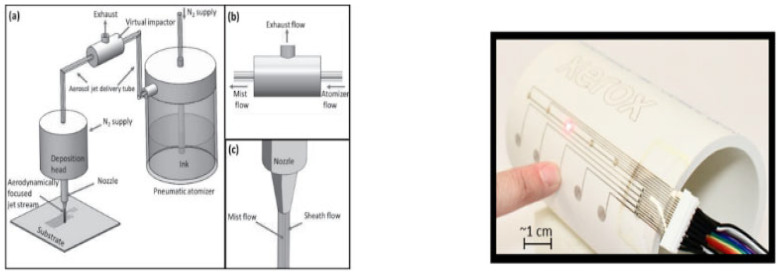
(**Left**): Aerosol jet printing working principle [53]. (**Right**): Printed hybrid electric capacitive touch sensor [54].

AJP involves three stages, specifically the atomization stage, aerosol delivery stage, and deposition stage. These three stages are displayed in Figure 7.

The process starts with the atomization stage where the formation of ink droplets or aerosols are formed. The ink is captured in an ink reservoir and secured in place in the pneumatic atomizer. This atomizer generates an aerosol of ink. By forcing ink through the nozzle at high speed, small ink droplets are produced. The atomization rate depends on the air flow of the nozzle and the ink viscosity [53].

After forming the aerosol, the transportation happens through a delivery tube to the deposition head. At this stage, the aerosol flow is aerodynamically focused by sheathing flow. By acting as a barrier for the aerosol ink to stick to the inner wall of the nozzle, the sheathing flow prevents clogging problems, which is visible in Figure 7 left. The nozzle-to-substrate distance is ca. 5 mm when the focused aerosol exits the nozzle. Due to the multi-axis motion of the deposition head and the build-in platform, the ink patterns are possible. Printing line after line is a time consuming technique, but this is a typical characteristic of direct writing techniques such as AJP [53].

A wide variety of materials such as metals, insulators, conductors, polymers, carbon-based materials, and biological materials can be used with the AJP technique [42].

AJP and the other discussed printing processes have a huge potential in making recently organic TFT’s from organic semiconductors and CNTs [42]. One of the latest achievements with the AJP technique are the printing of biomaterials and flexible and stretchable electronics. AJP also has several limitations such as the overspray, despite the good reproducibility and the high print resolution. Overspray is described as minimizing edge profile instability of the printed lines. Like in every printing technique, the optimization of process parameters for each ink is a time-consuming activity, mostly depending on the combination of the substrate–ink compatibility [29].

Table 6 shows the most important AJP specification characteristics and their scale.

AJP represents a capable extension of inkjet, in terms of printing resolution, viscosity of ink, and the distance from the printhead to the substrate. AJP offers several additional potential advantages compared to inkjet, particularly in the area of microelectronics and large-area, flexible electronics that can used for wearable applications. However, it is worth noting that the technology of aerosol printing is a younger technology compared to inkjet printing—the 2000s and 1950s, respectively. Consequently, inkjet printing is a more advanced technology, and there are more opportunities on how to use inkjet printing. Additionally, AJP is significantly more expensive than inkjet printing.

At the moment, AJP is catching up with inkjet printing due to its wide material range and high print standoff distance [54]. When you look for state-of-the-art applications, a functional capacitive sensing device is a good example. This device has five touch points, fabricated on the curved surfaces of polyvinylchloride and polycarbonate piping. The difficulty of this hybrid printed electronic device is in the printing on a three-dimensional surface, which require multidisciplinary knowledge of integrating and functioning of electronics [54].

An additional example created via AJP is the hydrogen gas flexible sensor made from nanowires. This sensor measures various concentrations of hydrogen gas. After coating the sensors in silver and silver–platinum, the sensors were put through several bending cycles. The outcome of these bending tests concluded that the silver–platinum coating reduced the flexibility of the sensor. The silver coating on the other hand has positive results after the bending tests [55].

#### 2.2.4. Practical Ink Related Limitations

Beyond intrinsic material properties, practical limitations related to the printability and long-term stability of functional inks must also be considered [56]. Rheological parameters such as viscosity and surface tension must be tailored for each printing technology, as deviations can lead to defects including coffee-ring effects, nozzle clogging, or incomplete line formation [57]. Long-term stability can be compromised by nanoparticle sedimentation, solvent evaporation, or binder degradation, which alter viscosity and cause batch-to-batch variation in print quality [58]. Furthermore, printed features may suffer from conductivity loss or adhesion degradation under thermal cycling, humidity, or UV exposure, particularly in outdoor or automotive applications [59]. Strategies such as adding dispersants, optimizing curing protocols, and applying barrier overcoats can mitigate these issues, but they often involve trade-offs between electrical performance, mechanical flexibility, and process compatibility [44,56].

## 3. Laser-Based 3D Electronics

Besides printing conductive tracks, it is also possible to create metal tracks on 3D substrates by laser processes and electroless plating.

### 3.1. Laser Direct Structuring (LDS) on Molded Substrates

#### 3.1.1. Introduction

**Figure 8:**
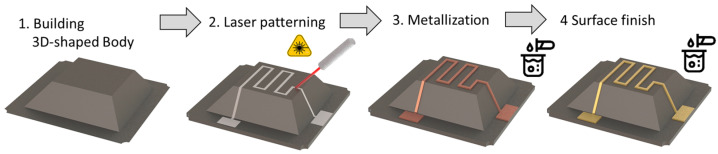
LDS process overview with the necessary steps to achieve a 3D circuit.

LDS is the most widely used 3D-MID technology [60]. Especially for miniaturization, design freedom, and flexibility in 3D geometry and track layout, the possibility of double-sided circuitry with vias, fine metal lines, and well-defined impedance structures draw interest on this technology. Figure 8 shows an overview of the LDS process. The conductive tracks are defined by a laser beam that activates selectively the surface of the 3D plastic part for subsequent electroless plating.

The first step is the 3D forming process of the basic body itself (1). Therefore, usually molding technologies like injection molding, compression injection molding [61], or in the case of thermoset materials, transfer molding or film-assisted molding [62] are used. The used plastic material is doped with a laser activatable additive consisting of a non-conductive inorganic compound. Meanwhile, a wide range of laser-structurable substrate materials is commercially available [63].

In the second step, the laser beam structures the desired circuit pattern (2). Laser structuring forms a micro-rough track, thereby activating the additive for subsequent electroless plating. A physical–chemical reaction produces the nuclei for the plating process. The micro-rough, “coral-like” surface structure enables a well-adhering metal layer [64].

Vias can be realized by laser drilling, which is carried out in the same process as the surface structuring. The via inside is activated as well as the 3D surface for electroless plating.

The third step is the metallization. Before that, cleaning of the part is necessary for removing the laser debris. Usually, wet chemical cleaning is applied. Alternatively, for high requirements regarding structure size also CO_2_ snow jet cleaning can be used [65].

Subsequently, the plating process starts with electroless copper deposition (3). The growth rate is about 5 µm/h [66].

Commonly, a surface finish of nickel and gold is then applied (4). Nickel serves as diffusion barrier and gold is a noble metal surface, which is versatile in use for different assembly technologies. Typical layer thicknesses are about 10 µm Cu, 5 µm Ni, and 0.1 µm Au. Some applications require special surface finishes like Ag or Pd/Au, which can also be applied. Based on a copper starting layer, copper electroplating can be carried out if a thicker layer is required [67]. However, due to the electrical contacting required for electroplating, the design of the circuit layout is limited.

The last step is the assembly of electronic components onto the conductive pattern. Many of the used plastics show high temperature resistance and are therefore solderable with standard surface mount technology (SMT) processes [68,69,70]. Depending on the substrate materials, for the assembly of electronic components, soldering with low-melting solder, isotropic conductive bonding, or wire bonding can also be applied.

#### 3.1.2. Substrate Materials

Depending on the application, important material characteristics such as processing temperature, mechanical and electrical properties, dimensional stability under heat, flowability as well as costs are to be considered [70]. Meanwhile, a wide range of LDS thermoplastic materials is available [63]. These materials were approved with cooperation between the compound suppliers and LPKF in terms of reliability, plating performance, and copper adhesion strength. Tengsuthiwat et al. [71] provides an overview of thermoplastic materials for MID substrates as well.

The available LDS thermoplastics can be divided into technical thermoplastics, e.g., ABS, PC, PBT, PPE, PA, and respective blends, and high-performance thermoplastics like e.g., LCP, PPA, and PEEK. The existing variety of substrate materials allows the user of the LDS technology to select suitable materials with unique properties to satisfy accurately the application specific performance needs. For example, PEEK is a thermoplastic semi-crystalline polymer with superior thermal performance but high cost. On the other hand, ABS is a low-cost material for applications where no high temperature resistance is needed.

Thermoset epoxy-based LDS materials [72] are used in particular when functionalized packaging solutions are required, e.g., a combination of encapsulation of chips with electrical circuits on the surface of the package [9,62,73,74].

There have also been advancements regarding the laser-activatable additives. Mainly copper chromite [75] and tin oxide-based additives [76] are used. The available types differ e.g., in color, seed-forming capability, purity, and mean particle size. One of these research topics addresses new additives, such as in [77], where researchers found that copper chromite can be substituted by copper aluminate. Additionally, copper aluminate supports the formation of conductive patterns with both thermoplastic and thermoset resins.

#### 3.1.3. Advantages and Disadvantages

The essential benefits of 3D-MIDs over 2D circuit boards arise from their proven capability to integrate both mechanical and electrical functions into just a single device. This includes the ability to create 3D circuit paths on non-conducting materials and less complex components that lower the size, weight, and assembly time of the device.

The LDS technology operates with a three-axis laser, which provides a high degree of flexibility and complexity. Therefore, this type of setup allows for full 3D capability by tracking the laser focus and rotating the components. High flexibility of the layout is given as the routing of the traces can be easily changed. Changing the circuit only means implementing new control data that are transmitted to the laser unit.

Furthermore, the technology enables the realization of conductor tracks with high precision. Depending on the material and the laser and plating parameter used, small line widths are possible and allow fine pitch applications. Line widths smaller than 50 µm have been achieved [65,78,79].

In production, the recommended line width is about 150 µm [69]. Thus, the weight and dimensions of the molded component can be noticeably reduced, enabling the miniaturization of MID [80].

All of these advantages prove valuable for prototyping. However, since injection-molded components require a mold, the production of injection-molded components for prototyping is comparatively high-effort and costly. More cost-effective and faster is the prototyping using additive manufacturing of the base body as described below.

Nevertheless, there are some limitations of LDS technology. Firstly, due to injection molding of the basic body, the LDS additive is needed for the entire component and not only on the surface. Secondly, the LDS technology requires a chemical plating process, which is paired with higher costs, especially for a bigger part size. Thirdly, depending on substrate material and laser parameter, the surface roughness of the metal layer can be increased. This can have an impact on subsequent assembly technologies using connectors and wire bonding of bare chips.

Compared to PCB, for LDS-MID, the number of circuitry layers is limited. LDS-MID typically have two layers, the front and back of the part connected with vias. Simple crosses can be realized by, e.g., passive SMD components such as resistors.

Taking into account the advantages of LDS-MID and PCB for each, hybrid approaches using both technologies enable additional benefits [81,82,83].

#### 3.1.4. Applications

LDS-MID are widely used in various industries, e.g., automotive, industrial, communication, and medical technology. Applications with integrated sensors, antennas, and lighting have been reported [84,85,86,87,88].

Other applications in medical technology are, for example, blood glucose meter, home healthcare, hearing aids, and lighting elements for dental application. Further applications can be found, for instance, in air-conditioning, safety technology with payment card readers, and the production of 3D interconnected packaging [68,89,90,91,92,93].

The potential of LDS-MID in space applications was successfully shown in a study within the ESA Artes 5.1 program [94]. Finally, millions of mobile phones use LDS-MIDs as space-saving integrated antennas [70,95,96].

Modern vehicles contain many sensors and electronic assistants, which serve to increase the comfort and safety of passengers. This multitude of electronics requires a reduction in the number of components and cost. Functions such as keys, plugs, and other connection elements can be integrated into an LDS-MID. By using LDS-MID, additional cabling can be omitted, the assembly outlay is reduced, and additional circuit boards can be saved. LDS-MID technology could be used to integrate the steering wheel controls into a more compact space [70]. In current research projects, the focus is also on structure-integrated electronics for wireless automotive applications, in which the antennas can be directly integrated into the plastic surfaces [97].

LDS-MID offer several advantages for high-frequency applications due to their 3D capability. Therefore, LDS MID technology is very interesting for such high-frequency applications, as numerous publications [98,99,100,101,102] illustrate. In this context, PEEK is a high-performance thermoplastic material, e.g., for use in applications for high-frequency technology. Due to its dielectric properties, PEEK has also the potential to substitute some fluorinated circuit board materials that are being discussed in connection with the PFAS ban. PEEK-based LDS-MIDs have been investigated, among others [61,103,104].

LDS is part of the process chain for the Ensinger Microsystems Technology (EMST), with which several sensors and microsystems can be fabricated [105,106,107,108]. The EMST process chain offers the possibility of building high-precision microsystems on LDS-capable TECACOMP PEEK LDS wafers without the need for lithography. It uses a combination of LDS for the leads of the sensors and physical vapor deposition and polishing for the sensor and other functional elements.

Combining LDS technology with laser plastic welding enables new possibilities for clean sealing and protection of LDS parts [108].

### 3.2. Laser Direct Structuring (LDS) on Coated or Additive Manufactured Substrates

For small quantities and prototypes, the manufacturing processes that require molds are often not economic. Therefore, the processes described below are suitable for prototypes as well as for individual small series. Another advantage is that the design and the process can be scaled directly into large-scale production.

#### 3.2.1. MID Lacquer

**Figure 9:**
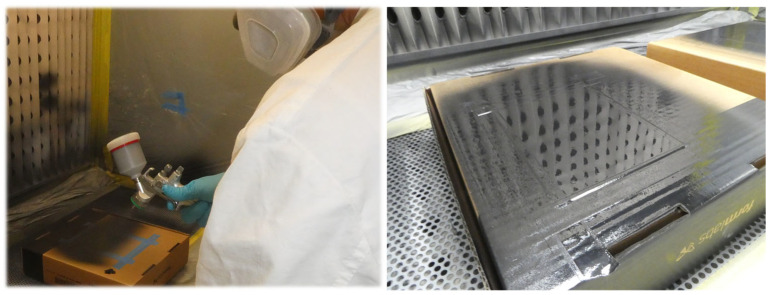
Processing of the MID lacquer using the HVLP technology.

In MID lacquer technology, an existing component is coated with an LDS-compatible lacquer [109]. The coating process was developed at Fraunhofer IEM and has been continuously optimized. The LDS-compatible lacquer is manufactured by Lackwerke Peters GmbH & Co. KG. The processing differs fundamentally from the well-known ProtoPaint LDS lacquer from LPKF [110]. ProtoPaint LDS is applied using an aerosol spray can. The result of this method is a coating of inconsistent thickness due to the non-constancy of the pressure within the can. In contrast, MID lacquer consists of three components—base coat, hardener, and thinner. This means that the properties of the material can be individually adjusted depending on requirements and prevailing climatic conditions. This enables the viscosity to be adjusted, ensuring optimal flow of the lacquer. The lacquer is vaporized using the high-volume low-pressure (HVLP) method at approx. 3.5 bar. The following Figure 9 shows the processing and the result of a high-gloss and uniform coating. Furthermore, the lacquer is very durable and still elastic. The lacquer effectively compensates for the different coefficients of thermal expansion (CTEs) between the substrate and the metallization.

Through the targeted pre-treatment of materials using primers or plasma activation, a wide variety of plastics, metals, ceramics, and glass can be coated. It has been demonstrated that the adhesive strength of the resultant layers is high; this can be attributed to the wet-on-wet process, which ensures excellent anchoring of the individual layers [111].

**Figure 10:**
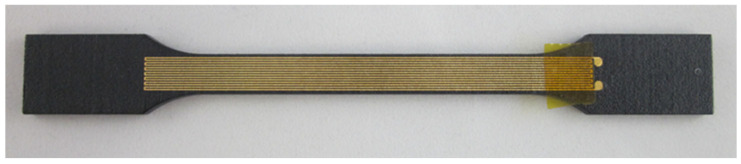
Strain gauge built on substrate using MID lacquer technology.

This technology can be used to functionalize existing assemblies, such as chassis components, by integrating sensors or actuators on the surface. In particular, the integration of sensor elements, such as strain gauges, can be implemented cost-effectively with this technology (Figure 10).

The production of MID lacquer components is a demanding process, as cleanliness and the right timing are essential for the quality of the produced components.

**Figure 11:**
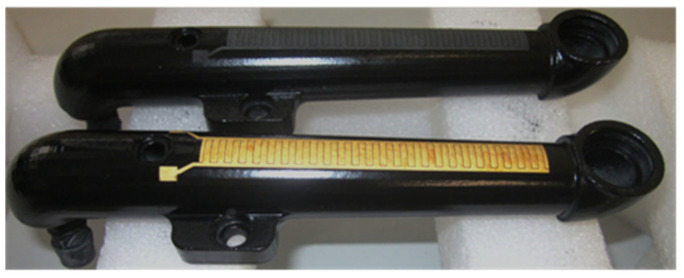
Laser structured MID (top) and metalized MID (bottom).

First, the components must be degreased, cleaned, and dried. Then, they are coated with a primer adapted to the base material. After a defined evaporation time, a filler is applied. After a further evaporation time, the final application of the MID lacquer is carried out. It is important that no dust can deposit on the lacquer; therefore, a special spray cabin is necessary. Following a further evaporation time for the tempering of the lacquer, the remaining solvents are evaporated, and the lacquer is hardened. Depending on the type of lacquer used, tempering too early can lead to orange peel. After a resting period of at least 12 h, during which the lacquer relaxes, the structuring can be carried out with a 1064 nm laser. The laser power required to activate the lacquer is approx. 4W, depending on the laser spot diameter. Figure 11 depicts an example of both the activated MID component (top) and the metalized component (bottom).

**Figure 12:**
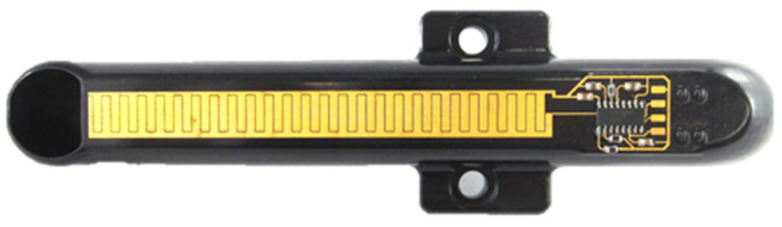
Dip pipe sensor with level detection electronic [109].

Figure 12 shows the corresponding application example where level electronics were integrated on the surface of a filler pipe. This component is required for a developer unit for newspaper rotary printing and significantly reduces assembly time by the all-in-one unit.

**Figure 13:**
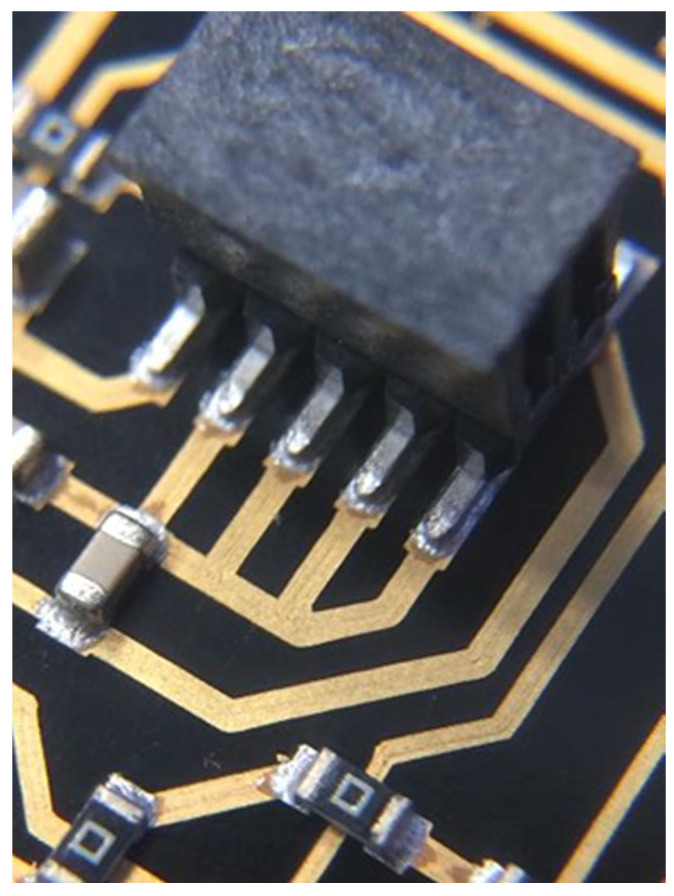
Electronic circuit built on a glass tile.

With the correct process, there is a strong adhesion of the various layers of lacquer to the substrate and to each other. Tensile and shear tests indicate a strength that is about 80% of that of a standard FR4 board. With special primers, lacquering of glass is also possible. Figure 13 shows an electronic circuit built on a glass tile. All components were soldered with tin-bismuth solder at 168 °C using the reflow technique.

#### 3.2.2. MID Resin and Stereolithography

**Figure 14:**
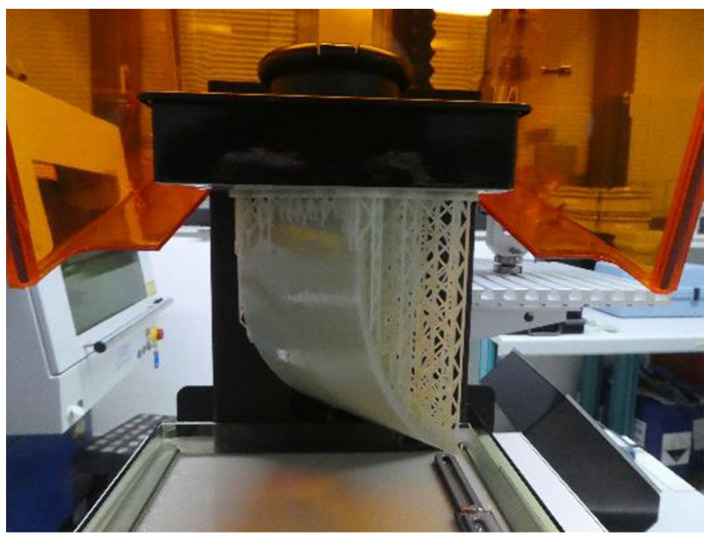
3D part printed with MID resin using SLA technology.

Stereolithography (SLA) with an LDS-modified MID resin is a tool-less process, where the prototype is available quickly and at low cost [11]. The MID resin developed by Fraunhofer IEM enables direct printing of an arbitrarily shaped MID component with the use of an SLA printer. The resin contains laser activatable additives and is suitable for the LDS process. It is important for the printer to have a wiper for the resin tank, so that the resin is continuously mixed in order to obtain a homogeneous dispersion. Figure 14 shows an MID substrate produced using SLA printing and MID resin.

**Figure 15:**
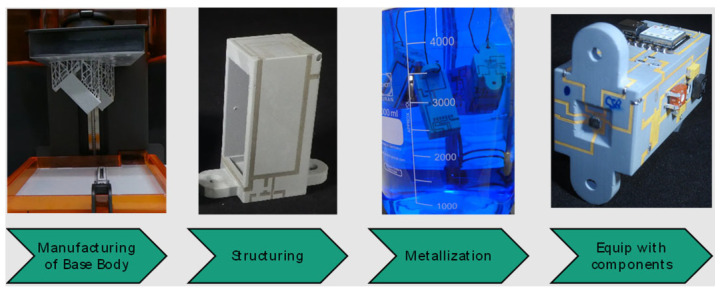
Manufacturing process.

After the printing process, the components must be cleaned and polymerized using heat and UV light. Afterwards, the component can be structured by applying a 1064 nm laser and subsequently metalized in an electroless plating process. This process is 100% compatible with the standard LDS process of molded parts. The MID parts produced in this way have a smooth and firm surface and can be fitted with SMD components and soldered in the reflow process. The following diagram (Figure 15) summarizes the process chain.

A similar approach using stereolithography was also presented by Piechulek et al. [112] and Contag [113].

#### 3.2.3. MID Resin and Hot Lithography

Hot lithography is an additive manufacturing process that produces industrial-quality, 3D-printed components. This is made possible by a heated printing process that processes viscous materials. LDS-modified resins are now available, which can be processed using hot lithography [114,115].

#### 3.2.4. Fused Filament Fabrication (FFF)

FFF is a widely used method for prototyping. A few materials are also available as LDS-containing filaments. For high-temperature applications, PEEK filament with LDS additives is available [116]. PC/ABS filament with LDS additives is also available, but due to the limited temperature resistance, the material is not suitable for soldering processes of electronic components, which limits the possible applications. Nevertheless, the material is very interesting for antennas, for example [117].

#### 3.2.5. Casting Silicone Substrates

First tests were carried out to deposit metal structures onto LDS-modified silicone, which can be casted into both planar 2D and 3D substrates [118]. Various structures such as horseshoe patterns, fractal meanders, and Hilbert inductors as well as a hemispherical 3D helical track were deposited and tested.

#### 3.2.6. Selective Laser Sintering (SLS)

A new approach combines selective laser sintering (SLS) with LDS and metallization [119]. For this process polyamide containing copper powder is used. The component surface is modified by laser structuring, so that copper powder is exposed, which serves as a seed for the subsequent electroless metallization.

### 3.3. Other Laser-Based Technologies

#### 3.3.1. Semi-Additive Process

The semi-additive process starts with a full-faced, electroless copper plating of an injection molded part. The isolations are patterned typically with an UV laser. After a cleaning step, which removes the laser debris to prevent electrical shorts, the copper layer is reinforced by electroless plating, e.g., with Ni/Au [81]. A similar technology called MIPTEC (microscopic integrated processing technology) was developed by Panasonic [120].

#### 3.3.2. SANCHO Process

For prototyping and small-scale production, various additively manufactured substrate materials can be functionalized using the so-called SANCHO process [121]. The acronym is derived from selective metal deposition on additively manufactured components using wet-chemical processes and laser-based surface modification. The process chain starts with masking the component’s surface and selectively removing the masking according to the circuit geometry using laser radiation. Subsequently, the surface is activated for electroless plating by using a wet-chemical palladium activator. Afterwards the masking is fully removed. Thereby, any excess activator is removed together with the masking. Finally, the metal layer is selectively deposited by electroless plating. This process also works with comparatively rough surfaces of additively manufactured substrates.

#### 3.3.3. Selective Surface Activation Induced by a Laser (SSAIL) [122]

The new SSAIL method is a promising technology for the fabrication of 3D electrical conductors on polymers. SSAIL is based on 3 main steps, as follows: laser modification of the surface, chemical activation of the modified surface areas, and electroless plating of the activated parts. The biggest difference to LDS technology is that this method does not require any additives in the polymer and can, therefore, be used for many materials.

## 4. Alternative Structuring Processes

In the past, other non-laser-based MID technologies were also used, although these have lost importance.

### 4.1. Two-Shot MID

Two-shot (or two-component) injection molding uses two different thermoplastic materials [64]. The conductor pattern is already structured during the injection molding process. This technology requires material combinations with different behavior towards the pretreatment and metallization process. This means that in an electroless metallization process, metal is deposited on one plastic component and not on the other. A core-catalyzed LCP is typically used as the metallizable component. In most cases, a non-core-catalyzed LCP is used as a non-metallizable component. Before electroless plating, the molded part is pretreated with a strongly alkaline solution.

Because the conductive pattern is already generated during the injection molding process, the process chain for two-shot MID is very short. However, the injection molds are very complex and are not flexible with regard to layout changes. Furthermore, the complexity of the 3D circuit, and the miniaturization of the circuit tracks is limited.

### 4.2. Hot Embossing

Hot embossing of MID is a process without wet processing steps [64]. The layout of an electronic circuit is milled into a steel stamp. An electrodeposited copper foil with suitable mechanical properties and with a cauliflower-like roughening on one side and a surface finish on the other side is placed with the rough side on a thermoplastic part. With a suitable press equipment, the foil is pressed on the thermoplastic part at an increased stamp temperature. The conductor tracks are punched out and firmly bonded to the thermoplastic substrate. Finally, the remaining foil is removed. The process is easy to use for simple 2D layouts on 3D plastic parts. In the past, rolled copper foils were also used. For such foils, a laser pre-cut is necessary due to their different mechanical properties [123].

### 4.3. FlameCon and PlasmaDust

There are two similar technologies known that apply the conductor tracks directly onto the part surface by spraying metal powder onto the surface—FlameCon and PlasmaDust.

FlameCon is a maskless thermo-kinetic application process developed by Leoni [124,125,126]. Metal powder is fed to into a chamber at a high temperature, melted, and sprayed under pressure onto the surface. The metallic structures adhere to practically any surface and can be used, for example, for the production of large parts to transmit signals without cables, e.g., in motor vehicles. Differently than electroless plating used in LDS and two-shot MID technology or printing technologies using inks, higher layer thicknesses up to 150 µm can be achieved in a single pass. Multiple passes are claimed to achieve coatings of more than 1000 µm. The line width of the deposited metal layers is in the millimeter range and, therefore, only suitable for macroscopic structures. Despite the many advantages, little is known by the authors about today’s industrial application.

Plasmadust is a maskless process that was developed by the company Reinhausen Plasma (now Relyon Plasma GmbH) [127,128]. It is similar to FlameCon but differs in the energy source. Low-temperature plasma is used instead of high temperature and pressure. The plasma gas is generated by means of pulsed arcing gas discharge of a metallic powder with grain diameter between 100 nm and 20 µm. The metal powder is fed continuously to the plasma. An atomizer/conveyor technology is needed to avoid agglomerates. The process allows coating of also temperature-sensitive plastics and can be used to apply metallic layers of copper, silver, tin, or even metal alloys and mixed systems. In combination with the adjustable, uniform particle flow, this ensures homogeneous and reproducible layer thicknesses up to 1 mm [127], but the technology is only suitable for macroscopic structures. The traversing speed can be up to 150 m per minute [127]. To the best of our knowledge, this technology is not widely used, however, and little information about reliability is accessible.

## 5. Connecting Rigid Components to a 3D Electronic Device

Both 3D and 2D electronic circuits need electronic components connected to each other by conductive tracks. How to create conductive tracks is explained in the previous sections of this paper. This section will elaborate on the connection of rigid components to the conductive traces on the surface of a 3D electronic device.

If the components can be placed onto the 2D substrate before shaping (e.g., by 3D forming) of the substrate happens, regular pick and place techniques for standard SMD components can be used. The applying of interconnection materials, e.g., solder paste and conductive adhesive, are similar to SMT on standard PCB.

When the components have to be placed after 3D shaping of the substrate, manual processes or advanced robotic systems are required to place the components. The biggest challenge is the placement of components on all sides of a 3D-shaped body. Traditional dispensing and placement machines are generally designed to work in 2D. These machines are tailored to planar PCBs, meaning they are not optimized for handling 3D substrates. In the past, machines suitable for the production of 3D-MIDs were developed by specialized machine builders. These machines were complex and expensive, i.e., they are often only cost-effective for large-scale production. However, there have been advancements in recent years for the economic-series and small-series fabrication of 3D-MIDs, especially through the development of more flexible and cost-effective machines, e.g., in [129,130]. There are also 5-axis systems for printed electronics on 3D substrates, which can be equipped with integrated pick-and-place modules [131].

Sufficient edge definition for the detection of fiducials is a prerequisite for automated placement. Referencing in opposition to the printing direction can pose a particular challenge. This can be a limiting factor, especially for digitally printed structures. This especially applies to 3D circuit carriers, where z-detection is a challenge in contrast to planar placement. For example, when transferring the connecting medium by dispensing, the needle height must be set very precisely, so that the dispensing quantity does not fluctuate too much.

In the following sections, reflow soldering, bonding with electrically conductive glue, and wire bonding are discussed as the most relevant assembly technologies regarding 3D electronics. Crimping, pressure sintering, and resistance welding will not be discussed in this paper, because this is not as easily usable for 3D circuit carriers.

### 5.1. Soldering Technologies

On standard 2D PCBs, reflow soldering is the most used technique for assembly of electronic components. However, this requires a comparatively high process temperature. Soldering can also be used for assembly of components on 3D electronic circuits, but there are a few extra things to keep in mind. Firstly, it is important to make sure that the heat is distributed evenly throughout the structure. When this is not the case, sensitive components may already be destroyed by too high temperatures, while other areas are not heated enough. Secondly, the accessibility of the components to be soldered can sometimes be challenging with 3D structures [132].

A further critical requirement for SMD assembly on three-dimensional circuit carriers is the fixation of components. Particularly for large, heavy components with a limited contact area, the adhesion forces provided by solder paste may be insufficient to prevent movement or complete slipping caused by acceleration during handling or on inclined surfaces due to gravitational forces. In such cases, the use of an additional surface mount adhesive may be required to ensure reliable component positioning [64,133]. However, for some components, this approach is unsatisfactory, because there is no suitable location to apply the additional adhesive.

**Figure 16:**
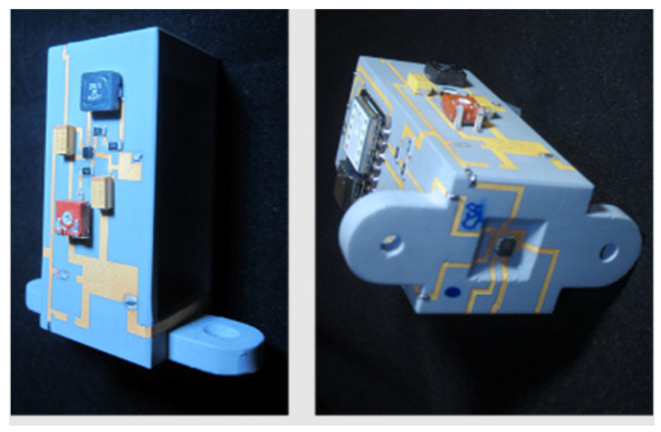
Active sensor that connects via Bluetooth as 3D MID with SMD on all six sides.

Figure 16 shows a 3D substrate with SMD assembled on all sides by soldering. A reflow process on all six sides is difficult, especially if heavy components such as inductors have to be mounted on the side surfaces or overhead. This problem can be solved by using solders with different melting points. Table 7 shows some of the commonly used solder materials that could be used for that.

First, the heavy or critical components are soldered with the solder that has the highest melting point. After that, the components on the other sides can be assembled with a low-melting solder, and so on.

SAC305 is widely adopted as the standard for lead-free electronics, since RoHS was implemented in 2006. It has a good wettability and acceptable mechanical properties. Ever since, a lot of alternatives have been investigated [134], but it remains the most used alloy in 2023 [135].

There are low temperature solder alloys like Indium and SnBi available and widespread on the market. Indium solder materials are available in different compositions and provide a high softness and ductility even at cryogenic temperatures. They are well-suited for joints between dissimilar materials under thermal cycling, as the solder accommodates stress through creep, maintaining joint integrity. Mechanical failure typically occurs due to stress overload or unidirectional creep, not thermal cycling. An overview of compositions, melting ranges, and failure modes of indium-based solders can be found in [136]. Indium-based solders are significantly more expensive than SnBi or SAC solder types, since pure indium is a rare metal. Therefore, it is mainly used in critical areas like cryogenics and hermetic sealing [137].

Another low-temperature solder alloy is SnBi. An overview of Sn-Bi compositions can be found in [138]. Sn-Bi eutectic solder has found broad application in wearable and flexible electronic systems due to its low processing temperature. However, its inherent limitations—including brittleness, poor drop impact resistance, limited flexural durability, and low mechanical toughness—must be addressed to enable reliable long-term performance.

The challenges vary depending on the underlying substrate technology:

- In the case of LDS-MID, the typically rough surface topography can result in the non-uniform or wide-spread wetting behavior of solder joints.
- In printed electronics, the tin in the solder tends to alloy with the thin silver layers, leading to intermetallic compound formation and the potential degradation of the conductive structures.

Common challenges can be delamination of pads and conductors because of insufficient adhesion strength between substrate and metal as well as cracks due to the thermomechanical load during the soldering process.

#### 5.1.1. Conveyor Oven Soldering

Conveyor oven soldering is the most common method of reflow soldering. First, the soldering paste is applied to the pads on the electronic circuit where the components need to be placed. Next, the components are picked and placed on top of the soldering paste. Finally, the assembly is put in a soldering oven, where it is gradually heated to the required temperature for the solder to melt. This temperature is highly dependent on the type of solder, cf. Table 5 [139,140].

For 3D components, the maximum clearance height during the reflow process must also be taken into account. Additionally, uniform heat distribution can be a limiting factor for 3D components.

#### 5.1.2. Vapor Phase Soldering

**Figure 17:**
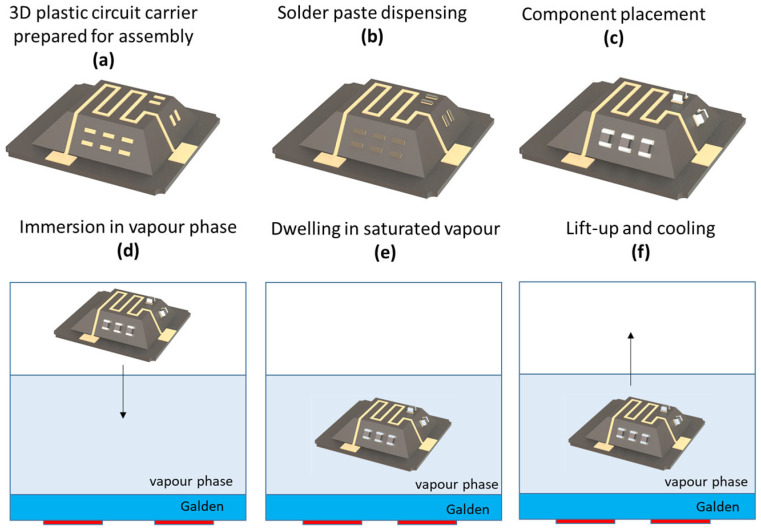
Vapor phase soldering process steps (**a**–**f**) loosely adapted from [141].

Vapor phase or condensation soldering is an advanced form of reflow soldering. This means that the soldering paste is also applied in advance. The main difference with traditional conveyor oven soldering is the way that the substrate is heated. Figure 17 shows the different successive steps (a–f) in a vapor phase soldering process.

The heat transfer fluid, also known as Galden fluid, is boiled and evaporates in a closed tank. When enough of the fluid is evaporated, the substrate is immersed in the vapor. The vapor then condensates onto the substrate surface. The condensation heat ensures uniform heating of the substrate, solder paste, and placed components. Once the assembly is hot enough, the solder paste melts, and the assembly is lifted out of the tank [141]. Because of the high heat transfer capabilities of the vapor, melting of the solder is more quick and even than in a normal convection reflow process [132]. This principle offers significant advantages, especially for 3D electronics, as the heating occurs uniformly over the entire component surface, independent of geometric design or mass concentrations.

However, the required temperature is still dependent on the type of soldering paste used. Different types of Galden fluid can be used with boiling temperatures between 150 and 260 °C [141].

#### 5.1.3. Laser Soldering

One of the main disadvantages of both conventional and vapor phase reflow soldering is the fact that the entire assembly needs to be heated above the melting point of the used soldering paste. This means that the entire assembly can just melt or can be damaged when it is heated for too long [141]. In contrast, laser soldering is known for its ability to only apply the heat to a very specific area. This means that the area surrounding the electronic components is not heated as much.

The most obvious downside of laser soldering for 3D applications is that in some cases, it might be impossible for the laser to reach the part on the assembly that needs to be soldered [142,143]. Furthermore, the sequential laser soldering process is time-consuming and, therefore, cost-intensive.

### 5.2. Conductive Bonding by Electrically Conductive Adhesive (ECA)

Another common technique is using electrically conductive adhesives (ECAs) to connect the components to the substrate. ECAs are widely used in electronic packaging applications [144]. They consist of a matrix that is responsible for the adhesion and binding properties of the compound [145]. The electric conductivity is generated by embedding a conductive filler in the matrix, which makes up approx. 80% of the total mass, e.g., silver, copper, or nickel powder. Mostly, the matrix consists of an epoxy- or acrylate-based resin.

Conductive bonding using ECAs is an efficient technology when a temperature-sensitive electronic element, which only needs a relatively small current, is connected to a circuit. ECA are suitable for low process temperatures in respect to low-melting substrate materials and temperature-sensitive components. However, especially for high-density BGA components or components that require a high current, such as the power supply, conductive bonding is not an alternative to the classic solder connection.

There are two primary categories of ECA based on the mechanisms of conductivity—isotropic conductive adhesives (ICA) and anisotropic conductive adhesives (ACA).

ICA are electrically conductive in all directions. One major disadvantage of ICA is that the cure time is longer than solder reflow times, but it can be shortened by “snap-cure” polymers. These polymers use catalysts and fast variable frequency microwave curing. The reliability of ECA assembled SMD under thermal cycling is measured to be better than the reliability of soldered SMD [146,147]. On the other hand, noble metal surface finishes of the SMDs are required, and moisture sensitivity might become an issue.

Anisotropic conductive adhesives (ACA) contain conductive particles that are mechanically clamped between the bond surfaces and are, therefore, just conductive in the Z-axis. There is no conductivity in the X- and Y-axis. Size and material of the conductive particles vary. Fine-pitch applications are possible, since short circuits are avoided. However, ACA requires reduced surface roughness compared to ICA. The advantages of ACA are the lower processing temperature and the simple processing. This lower temperature increases the potential breadth of anisotropic conductive adhesive applications and facilitate faster manufacturing and assembly through-put and overall lower cost [148].

### 5.3. Chip Assembly by Wire Bonding [149,150,151]

With wire bonding, electrical interconnections between bare silicon or other semiconductor chips to the substrate can be created. First, usually isotropic or non-conductive adhesive is used to fix the die to the substrate, and wire bonding is conducted afterwards. It uses bonding wires made of highly conductive materials such as, e.g., gold, aluminum, or copper with a thickness of usually between 15 and 75 µm. There are two main variants of this process, including gold ball thermosonic bonding and aluminum wedge ultrasonic bonding.

For gold wire bonding, the first step is the generation of a gold ball at the end of the wire by a small electrical discharge. After formation of the ball, it is placed on the components’ bond pad, and subsequently, pressure, heat, and ultrasonic forces are applied at the other end of the wire to form a metallurgical weld. The main parameters here are the heating time, the force magnitude, and the heating power. For aluminum wire bonding, a clamped aluminum wire makes contact with the bond pad. While ultrasonic energy is applied for a certain amount of time, which forms the first wedge, the wire is pressed against the corresponding lead finger. Additional ultrasonic energy forms the second bond, after which the wire is broken off by being clamped and moved.

In general, a substrate roughness Rz lower than 10 µm is required for wire bonding. Therefore, wire bonding on laser-based 3D electronics can be challenging.

For LDS technology, appropriate substrate roughness can be obtained using the suited laser structuring parameter and CO_2_ snow jet cleaning [152]. Extensive scientific studies have been conducted on fine wire bonding on thermoplastic LDS-MIDs made from LCP [153]. In this context, the stitch-on-ball method also demonstrated a stable process fulfilling the DVS bulletin. In addition, products manufactured using wire bonding already exist [154].

Regarding IME, to the authors’ knowledge, no concrete applications in commercial products currently exist, as wire bonding on IME poses significant challenges. The extremely thin silver structures are prone to degradation during the bonding process. Moreover, the implementation of 3D structures presents considerable process-related challenges as like with LDS-MID. In ball-wedge bonding, the bond surfaces must be heated, and the components require stable support points beneath the bond site to effectively implement the bonding energy. Consequently, the technology readiness level is assessed to be very low.

## 6. Comparison on Suitable Combinations of Substrate and Interconnection Technology

### 6.1. Comparison and Overview of 3D Plastic Circuit Carrier Technologies

Table 8 provides an overview of 3D plastic circuit carrier technologies and various evaluation criteria. Given that the suitability [155,156] of each technology is influenced by a multitude of factors, the table does not claim to provide a comprehensive assessment.

The table reveals that, e.g., aerosol jet and inkjet printing and LDS on thermosets are well-suited for fine-pitch applications, whereas other processes offer a lower resolution but a significantly higher current-carrying capacity (e.g., hot embossing, valve jet printing). Inkjet and aerosol jet printing, by comparison, are more precise than screen printing with a better resolution. Due to the higher layer thickness laser-based MID offer a higher current-carrying capacity compared to print-based MID.

The 3D capability varies considerably; while most laser-based MID technologies exhibit few limitations with respect to shaping processes (e.g., injection molding design rules), IME technologies are significantly constrained due to the 3D forming process by thermoforming and the limited stretchability of the conductive structures. Also, print-based MID are limited regarding 3D capability due to the short distance from nozzle to surface (print standoff distance) and the jetting head geometries that are commonly flat.

As laser structuring and jet printing are digital processes, layout changes are easy to realize.

A wide variety of substrate materials is available for almost all of the described technologies. However, LDS necessitates the presence of specific additives in the substrate materials, while IME relies on highly thermoformable semi-finished products, both of which significantly constrain material selection. Many materials used in LDS are high-performance thermoplastics that have a high thermal stability and support reliable soldering, making them suitable even for high-temperature applications. Most materials used for IME are industrial or engineering plastics like PC and PET [18] and have a lower thermal stability on the other hand. Print-based MID offers a wide range of material selection. Only the thermal requirements of the metallization processes need to be considered—both the sintering temperature during the process and any potential post-sintering effects, such as unintentional electrical sintering observed in IME [177].

With LDS (applicable to both thermoplastics and thermosets), vias can be generated directly during manufacturing, enabling the implementation of two conductive layers on the substrate side without significant challenges. In print-based MID, for conductor crossings, the deposition of insulating layers is conceptually feasible with inkjet [178,179,180,181]. Furthermore, ICA can also be utilized for the realization of vias. For IME, to the authors’ knowledge, such an approach has not been demonstrated, potentially due to constraints imposed by the thermoforming process.

Laser-based MID are well-suited for high-frequency applications. In contrast, IME and print-based MIDs exhibit limitations in this area due to the comparatively low and non-uniform electrical conductivity of their conductive structures.

No general statement can be made about the scalability of the technologies, as it depends on numerous factors. However, the following generalized statements can be made:

- All processes are batch capable, if the 3D geometry allows it.
- IME is best suited for large-area circuits, as the conductive pattern can be rapidly applied in 2D using screen printing of pastes. Accordingly, the associated applications typically involve large-area components.
- In laser-based and print-based MID technologies, structuring is carried out sequentially using laser or jetting technologies, respectively. As a result, the process is comparatively slow for large circuits on large surface areas.
- Laser-based MID technologies are well-suited for miniaturized 3D parts, as the electroless plating process can be carried out by barrel plating.
- The nozzle-to-surface distance in the aerosol jet process is less critical than in inkjet or valve-jet technologies. This makes it easier to follow 3D surfaces with the jetting head without multiple clamping setups.
- In 2D screen printing, valve- and inkjet printing can be very cost effective and scalable while in 3D it presents significant challenges.

The next few years will be crucial for 3D electronics to reach commercial maturity. Reducing the costs of individual technologies is the key to sustainable growth and consolidation in this area.

### 6.2. Technology Combinations Considering Substrate Temperature Stability

Many combinations of substrate and interconnect technologies can generally be considered unsuitable due to the thermal limitations of the substrate material compared with the required interconnect processing temperatures. Table 9 provides a selection of possible substrate materials, regardless of the technology used. Most IME materials can, therefore, be ruled out from the outset and only be considered with low-melting solder. Also, not all LDS materials can be processed using SAC solder.

**Figure 18:**
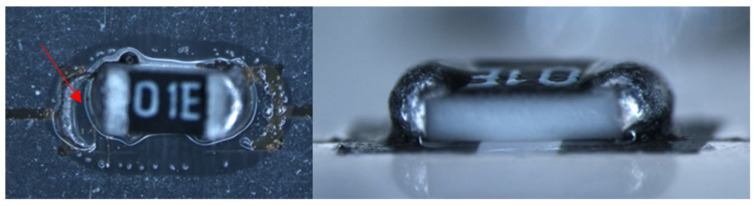
Dissolved Ag printed pad after soldering process (**left**). Poor wettability on Ag layer deposited by aerosol jet printing (**right**). Area in which the solder has dissolved (red arrow).

In addition, many of the print-based electronics are not suitable for soldering processes, because the metallization layer (usually Ag-based) dissolves into the molten solder (s. Figure 18, left). Since the diffusion rate depends on the material combination and temperature, and the diffusion zone depends on the product of diffusion rate and time, it is still possible to find process windows that do not lead to complete alloying [194]. An initial estimation of feasibility may be derived from diffusion curves available for selected material combinations [195,196,197,198]. Another issue with printed electronics can be a poor wettability with some inks [199,200] (s. Figure 18, right).

### 6.3. Isotropic Conductive Adhesives (ICA) on Print-Based 3D Electronics

Isotropic conductive adhesives (ICAs) are an emerging and promising solution to the lack of traditional soldering compatibility with (silver) printed electronics caused by silver diffusion and substrate temperature limits. ICAs are polymer-based (mostly epoxy-based) materials filled with conductive particles, typically silver or nickel between 20% and 35% in volume, enabling electrical and thermal conductivity in all directions. These ICAs have low-temperature processing properties in the range of 80–120 °C, which makes them ideal for use on plastic substrate carriers such as PMMA and PC. Unlike anisotropic conductive adhesives (ACAs), ICAs do not require directional alignment, simplifying the deposition and assembly process on complex surfaces or with complex components. The main challenge of ICAs include achieving sufficient conductivity with minimum filler loading, while maintaining excellent flexibility, adhesion, and long-term reliability under mechanical and thermal cycling. ICAs can be deposited with various techniques such as stencil printing, direct writing, or even screen printing. ICAs can also be used to create vias in multi-layer of multi substrate interconnections [201,202,203,204].

### 6.4. Soldering on Laser-Based 3D Electronics

Soldering with tin–silver–copper (SAC) alloy is the standard method for mounting SMD components on LDS-MID. This widely adopted PCB-based process is well established across the supply chain. However, the solderability depends on the properties of the LDS substrate material and their metallization. Some common LDS substrate materials that are generally solderable are PPA, PPS, PEEK, and LCP (cf: Table 9).

Substrate materials like, e.g., PC, PBT, and PPE are in principle solderable with low temperature solder alloys based on Indium or SnBi. Soldering with those alloys is less common but generally follows the same principles as SAC soldering. Key differences relevant to their use include maximum operating temperature and mechanical durability.

LDS-MIDs with SAC soldered components are used in a wide range of applications (see Section 4) and have been qualified for these purposes. Numerous studies on LDS-MIDs and reliability assessments have been conducted to support this. For example, in [205], a reliability qualification of soldered SMD components on LCP and PA6/6T substrates was conducted for automotive applications within a temperature range of −40 °C to 125 °C. The evaluation was carried out in accordance with IPC-9701 and JEDEC JESD22-A104D standards and included a comparison with conventional FR4 substrate materials.

SnBi alloys are used less frequently than SAC-based solders, and accordingly, data on their reliability are more limited. However, due to their mechanical properties, SnBi alloys provide lower reliability than SAC alloys, especially regarding thermo-mechanical stress [206]. Based on homologous temperature, the operating temperature for SnBi can be estimated at around 80 °C, using the homologous temperature of SAC alloys at 125 °C as a reference [207].

For MID, often, vapor phase soldering is preferred, as it allows for a uniform temperature distribution. In general, high surface roughness and the characteristics of the conductive tracks may result in significant solder material spreading [64]. Owing to the three-dimensional aspect, the substrates are often bulky. Tempering prior to soldering can reduce the risks of delamination and cracking of conductor traces during thermal cycling in the soldering process.

When using polyamide substrate materials, moisture absorption must be carefully managed in addition to ensuring reliable processing during the reflow soldering process.

### 6.5. Comparison of Reliability

Given the wide range of 3D electronic application areas, the requirements for their quality and reliability vary accordingly. A general definition of the load limits is often not feasible due to their high degree of individuality. Furthermore, there is a lack of sufficient knowledge regarding technology-specific factors influencing reliability, as well as a comprehensive understanding of the complex interactions that lead to failures. Therefore, the following section focuses on the state-of-the-art of the most common material–technology combinations.

#### 6.5.1. Reliability of Print-Based Substrates

There are several publications on the reliability of printed substrates. However, comparability is difficult due to the wide variety of combinations of substrate material, ink, sintering methods and profiles, passes, and much more. Therefore, only some general findings are outlined in the following.

- The mechanical stress in the inkjet-printed circuit boards strongly depends on the sintering process because of its impact on density and modulus, shown, e.g., in [208,209]. During subsequent thermal cycling, the stresses in inkjet-printed structures are relatively comparable to FR4 PCB. However, inkjet-printed circuit carriers show noticeably greater deformation than FR4 PCB.
- ICA materials on inkjet-printed circuit carriers can offer a similar reliability as on traditional PCB [210]. The failure can be a crack located between the SMD component interface, which is traced back to the high CTE mismatch between the ICA and the SMD component and the mechanical stress that this induces [211].
- The authors of [212,213] showed that the SMD size and the CTE of the substrate are critical properties, which need to be considered for highly reliable connections that need to withstand thermal load. Furthermore, it was demonstrated that, for adhesively bonded SMDs, a characteristic fatigue life of approximately 3500 cycles under thermal cycling between +125 °C and −40 °C, as well as more than 1000 h under combined temperature–humidity stress at 85 °C and 85% relative humidity, can be achieved for various SMD sizes.

It can be summarized that many fundamental reliability aspects are shared with conventional PCB [211]; printed electronics are additionally influenced by process- and material-specific factors. These include printing resolution and uniformity, ink formulation and stability, substrate–ink adhesion, sintering quality, layer thickness and porosity, as well as resistance to environmental influences [209,214]. Consequently, the critical parameters differ significantly due to the distinct manufacturing methods, material systems, and often more flexible or unconventional application scenarios, although the underlying principles of reliability remain similar.

#### 6.5.2. Reliability of In-Mold Electronic

In-mold electronics (IME) is still an emerging technology but has been the target of several studies. Yet, only a few references regarding the reliability are available. Since companies like TactoTek Oy [215], LEONHARD KURZ Stiftung & Co. KG [216], and others brought the technology in multiple applications, especially automotives, it can be concluded that the technology is capable of achieving high reliabilities.

Recent studies have systematically investigated the long-term reliability of IME under environmental and mechanical stress. Accelerated aging tests show that exposure to elevated temperature and humidity can induce degradation in printed conductive traces and interconnects, primarily due to moisture ingress and thermal expansion mismatch between printed layers and the polymer substrate [217,218]. Further, Lall et al. examined the reliability of additively printed IME circuits using electrically conductive adhesives (ECA) under sustained high-temperature operation (up to the level of thermal stress seen in harsh operating environments), identifying resistivity drift and failure mechanisms linked to prolonged exposure and thermo-mechanical cycling [3]. Cyclic bending and thermo-mechanical fatigue can lead to crack initiation at the interface of printed conductors and the substrate, especially in areas with sharp curvature or around embedded components. A broader technology review by Beltrão et al. summarizes typical failure modes in IME—such as cracking of printed traces due to molding strain, adhesion loss, and moisture-induced degradation—and emphasizes the need for ink and substrate systems tailored for thermoforming and injection molding [219]. To ensure reliable IME performance in demanding environments, design-for-reliability approaches—including material selection (low CTE inks, barrier coatings), process optimization (controlled molding temperatures, gradual deformation), and environmental sealing—are essential.

Further, ref. [219] focused on the reliability of in-mold flexible electronic systems in the face of challenging automotive environments. A drop of resistance could be seen in the conductor path during high-temperature operating life after 480 h at +85 °C but remained minor compared to the limit of 20% according to IPC-9701A standards.

Issues arise primarily within the IME process itself, e.g., ink wash-off the film during overmolding from if the shear stress is higher than the adhesion between the ink and the film. A summary regarding this can be found in [23]. The temperatures during process, mold, and melt are the main reasons for ink failure. Other defects can be torn films, e.g., close to the corners of the mold. Lower injection velocity helps to prevent the ink wash-off, but on the other hand, part warpage is more critical.

#### 6.5.3. Reliability of LDS-MID

The reliability of LDS-MID is crucial, particularly in safety-relevant sectors, such as automotives and medical technology. A comprehensive survey conducted among companies along the MID value chain in 2016 showed that reliability (“higher failure frequencies”) is considered one of the main challenges due to the lack of a standards [220]. However, a lot of research addressed this topic [205,221,222,223]. LDS-MID require a more considered approach to technical design due to their often less favorable thermal expansion coefficients and high range of materials compared to FR-4. Adding to the difficulty, some LDS thermoplastics show anisotropic behavior. The following failures may occur if the design is unfavorable, as follows:

- Cracking of LDS conductor tracks under thermal loading, e.g., in [205,222];
- Delamination-induced failures due to insufficient adhesion strength, e.g., in [205,222];
- Creep fracture in the solder [205];
- Mixed fracture [205].

The authors of [222] investigated measures to improve metallization adhesion strength and to prevent conductor track cracking under thermal stress. The results clearly demonstrate position-dependent differences on a 3D substrate. Notably, soldering-induced stress has a significant impact on the failure behavior of the test circuits. Conductor tracks over welding lines of injections molded parts exhibit the highest tendency for crack-related failures. Convex surfaces also show an increased risk of failure. In contrast, ejector pin marks and concave surfaces were less affected by cracking. To prevent conductor track cracking, the design rules for LDS-MID components should be strictly followed [69,224].

LDS-MID substrates, as mentioned with less favorable thermal expansion than FR-4 and sometimes anisotropic properties, required extensive testing and modeling to ensure solder joint reliability. In this context, in [205], the reliability of soldered SMD components on LDS-MID under thermal cycling based on JEDEC JESD22 for different thermoplastic materials is investigated—PET + PBT, PA6/6T, and LCP. Selected components are soldered with SAC305 and tested in temperature shocks from −40 °C to 125 °C. LCP performed better than PA6/6T and FR4. Plastic SMD components showed better performance on LDS-MID substrates than on PCB. This can be explained by the fact that LCP exhibits a coefficient of thermal expansion (CTE) similar to copper in one spatial direction. On the other hand, ceramic resistors demonstrated a longer lifetime on PCB, which is attributed to the smaller difference in CTE. Therefore, reliability can be improved by choosing the appropriate substrate, component size, and lead design. It has been shown that for flat 2D applications, a thermo-mechanical model description of LDS-MID substrate materials is sufficient. For 3D applications, however, coupling with injection molding simulation data, in combination with a thermo-elastic material model is recommended.

Without the use of solder resist, the application of solder paste results in widespread wetting of the metallization. This can cause the solder to flow out of the intended joint area, leading to inhomogeneous solder joints with poorly formed solder menisci, which in turn can negatively affect the long-term reliability of the assembly [225].

The authors of [221] demonstrated that the correct material selection and proper orientation are important as well. Furthermore, it was shown that protection mechanisms like overmolding, potting, or coating improve the reliability in environmental testing; however, even unprotected parts showed good results. The assessment included thermal shock testing, drop testing, and the evaluation of the system’s resistance to media ingress.

The authors of [226] analyzed the influence of different assembly variants on the reliability of MID and showed that NCA and ICA are comparable.

The authors of [223] investigated conductor track defects, identified their root causes, and assessed electrical performance through conductivity measurements. The analysis revealed that specific combinations of substrate materials and metal layers offer significant improvements in production quality, enabling LDS-MIDs to maintain performance under harsh operating conditions. Almost all failures occurred at the solder joint. Especially, two parameters were identified as having a decisive impact on performance—the type of thermoplastic substrate material and the heat input during soldering. It was revealed that a lower nickel layer thickness in the metallization stack is better. In addition, surface roughness has a huge impact on the reliability. Lower roughness results in more failures [222,223,227].

The authors of [228] showed that increased surface roughness combined with reduced nickel layer thickness has the most significant positive impact on the reliability of the conductor tracks. Additionally, increased phosphorus content in the nickel layer further enhanced reliability, since an increased phosphor content improves ductility. Consequently, nickel-free metallization systems were investigated in [229]. Depending on the substrate material used, some nickel-free metal layer stacks exhibited performance comparable or significantly superior to the standard Cu/Ni/Au metallization. Cu with EPIG (electroless palladium immersion gold), direct immersion gold, and immersion silver finish consistently showed good results, while Cu with OSP and Cu with immersion tin finish is not recommended.

In summary, LDS-MID are reliable when design rules are followed and approved LDS material and suitable metallization are used. This is further demonstrated by the numerous applications, where the technology has been deployed or certified.

## 7. Conclusions

This review provides a comprehensive overview of state-of-the-art materials and technologies used to realize electronic circuits on 3D plastic carriers—commonly referred to as 3D electronics. Selecting the appropriate combination of technologies is highly application-specific and depends on factors such as design complexity, required structure size, production volume, and material constraints.

It can be concluded that there is no universal solution but that the optimal choice depends on several interrelated factors. These criteria must be weighed carefully in order to identify the best-fit combination of technologies and materials for a given use case.

To support the selection of suitable methods, each section explained the working principles, materials, and typical application areas of the respective technologies. Comparison tables were included to enable straightforward evaluation of deposition techniques, 3D forming processes, and interconnection options, offering a practical guide for engineers and researchers.

With regard to IME in the context of printed circuitry on initially 2D substrates, screen printing is most widely used compared to other printing techniques. It can be used with most substrates and many conductive inks that are available. Screen printing and thermoforming is also highly scalable. However, the ink formulation for thermoforming remains challenging. IME are cost-efficient and highly scalable. While screen printing is the state-of-the-art, inkjet printing in the field of IME is still in research. So far, the requirement for nano-sized particles and low deformation are disadvantageous for flexible and stretchable inks [23].

With print-based MID, the circuitry is applied on an already 3D-shaped substrate body. Material deposition can be carried out by different printing techniques that all have their advantages and disadvantages. The conductive inks used in the various droplet-based printing techniques can be divided into three main categories—metal nanoparticle inks, carbon nanotube inks, and organic inks. Different substrates can also be used across multiple printing techniques, with some restrictions due to high temperatures or specific process parameters. Valve jetting is comparable to inkjet printing, but an important advantage is that jetting valves can operate with higher accuracy and at high speeds. Aerosol jetting is a newer, more precise, and higher resolution alternative for inkjet printing. However, it is significantly more expensive.

While providing more complexity, laser-based 3D electronics, especially LDS-MID, offer a large degree of flexibility on top of enabling a more advanced miniaturization than IME or print-based MID. Recent developments have produced an increased number of available substrate materials with different properties, which can be selected for many applications. Using additive manufacturing for the base bodies together with laser-based substrate technologies enable ways for prototyping.

The assembly and interconnection technology for each circuit carrier must be evaluated on a case-by-case basis. The simplest selection criterion is the temperature stability of the substrate materials. However, due to the significant variation in thermal properties even within a single thermoplastic or polymer class, universally applicable statements are difficult to make. It is, therefore, recommended to refer to the specific material data sheet. Other factors, such as alloying behavior of the surface metallization and solder, may also play a role, and the challenges vary depending on the underlying technology, as follows:

- In the case of laser-based MID, the typically surface topography can influence the wetting behavior of solder joints.
- In printed electronics, the tin in the solder tends to alloy with the thin silver layers, resulting in the formation of intermetallic compounds and possible degradation of the conductive structures.

The present evaluation cannot relieve the user of the task of selecting the ideal combination of substrate and interconnection technologies for a specific application. However, it reveals trends, indicating which combinations offer advantages based on the criteria, which is helpful for selecting suitable combinations.

## Figures and Tables

**Table 1:** Typical polymer types for IME [13].

Polymer Type	Typical Molding Temperatures
PP (polypropylene)	190–220 °C
PC (polycarbonate)	260–340 °C
PET (polyethylene terephthalate)	250–260 °C
PMMA (polymethyl methacrylate)	240–280 °C
TPU (thermoplastic polyurethane)	190–210 °C

**Table 2:** Functional inks [23].

Conductive	Semiconductive	Dielectric
Ag, Cu, Ni	OSC	SiO_2_
PEDOT:PSS	P3HT	PVA
CNT	PQT	PVP
Graphene		PMMA
ITO		Epoxy

**Table 3:** Screen printing parameters [12,22].

Parameter	Scale
Print resolution	30–100 µm
Print thickness	3–30 µm
Printing speed	9.96–1660 mm/s
Solution viscosity	0.500–5 Pa·s
Solution surface tension	38–47 mN/m

**Table 4:** Valve jet printing parameters.

Parameter	Scale
Print resolution	5–100 µm
Print thickness	>100 µm
Printing speed	100 dots/s
Solution viscosity	100 Pa·s
Solution surface tension	20–40 mN/m

**Table 5:** Inkjet printing parameters [12].

Parameter	Scale
Print resolution	15–100 µm
Print thickness	0.01–0.5 µm
Printing speed	5.48–1377.8 mm/s
Solution viscosity	0.001–0.10 Pa⋅s
Solution surface tension	15–25 mN/m

**Table 6:** Aerosol jet printing parameters.

Parameter	Scale
Print resolution	>85 nm
Print thickness	10 nm–5 µm
Printing speed	200 mm/s
Solution viscosity	0.001–2.5 Pa·s
Solution surface tension	10–20 mN/m

**Table 7:** Some solder materials for 3D electronics.

Solder Type	Composition	Melting Point
SAC305	96.5Sn/3Ag/0.5Cu	217 °C
SnBi (Ag)	42Sn/57Bi/1Ag	138 °C
Indium	52In/48Sn	118 °C

**Table 8:** Overview and evaluation of 3D plastic circuit carrier technologies.

		Minimum Pitch [µm]	Minimum Line Width [µm]	3D Capability	Layout Changes	Ampacity	Vias	Source
**IME**	**Inkjet printing**	20	10	−/+	++	−	−	[12,157,158,159,160,161]
	**Screen printing**	50	30	+ *	−	−/+	+	[12,53,161,162]
**Print-based MID**	**Valve jetting**	n/a	400	−	++	++	+	[163,164]
	**Inkjet printing**	20	10	−/+	++	−	−	[157,158,159,160]
	**Aerosol jet printing**	20	10	++	++	−−	−	[161,165,166]
**Laser-based MID**	**LDS**	70	50	++	++	−/+	+	[79,93,167]
	**LDS thermoset**	60	30	++	++	−/+	+	[10,168]
	**MID resin**	100	100	++	++	−/+	+	[11]
	**MID lacquer**	60	60	+	++	−/+	−/+	[169,170,171]
**Alternative MID**	**Hot embossing**	300	400	−	+/−	++	−	[64,172]
	**2-Shot MID**	150–250	150–250	+/−	−−	−/+	+	[173,174,175]
	**FlameCon and PlasmaDust**	>200	n/a	+	+	+	−	[64,125,126,128,176]

* After 3D forming process by thermoforming.

**Table 9:** Substrate materials considering temperature stability. Feasible (✔), not always feasible (o), and not feasible (x).

Substrate Material	Shape Resistance/DTUL HDT A (@1,8 MPa) [°C]	Tmelt [°C]	Source	Compatible with SnBi Soldering	Compatible with SAC Soldering
ABS	80–105	130	[182,183,184]	x	x
ABS (TF)	101	-	[182]	x	x
LCP	<250	320–325	[183]	✔	✔
LCP (LDS)	221–274	310–335	[185,186]	✔	✔
PC	120–135	148–230	[183,184]	o	x
PC (TF)	127	-	[187]	o	x
PC (LDS)	86–103	-	[188,189]	o	x
PMMA	75–105	110	[183,184]	x	x
PMMA (TF)	83–105	-	[190]	x	x
PETG	65	100	[183,184]	x	x
PEEK	152–280	340–345	[183,184]	✔	✔
PEEK (LDS)	255	343	[191]	✔	✔
PPA	307	315	[192]	✔	o
PPA (LDS)	290	-	[193]	✔	o
PBT	50–65	220–225	[183,184]	o	x

## Data Availability

All data used are shown in the text.

## Abbreviations

The following abbreviations are used in this manuscript:

3D	Three-dimensional
ACA	Anisotropic conductive adhesives
AJP	Aerosol jet printing
AgNP	Ag nanoparticle
AgNW	Ag nanowires
CNT	Carbon nanotube
DoD	Drop-on-demand
ECG	Electrocardiogram
ECA	Electrically conductive adhesives
EGaIn	Eutectic gallium indium
EL	Electroluminescent
EMST	Ensinger Microsystems Technology
ESA	European Space Agency
FDM	Fused deposition modeling
FFF	Fused filament fabrication
HTOL	High-temperature operating life
HVLP	High-volume low-pressure
ICA	Isotropic conductive adhesives
IME	In-mold electronics
ITO	Indium tin oxide
LED	Light-emitting diode
LDS	Laser direct structuring
MID	Mechatronic-integrated devices
MIPTEC	Microscopic-integrated processing technology
MOD	Metal–organic decomposition
OLED	Organic light-emitting diode
OSC	Organic semiconductor
P3HT	Poly(3-hexylthiophen-2,5-diyl)
PBT	Polybutylene terephthalate
PC	Polycarbonate
PCB	Printed circuit board
PE	Printed electronics
PEDOT:PSS	Poly(3,4-ethylenedioxythiophene) polystyrene sulfonate
PEEK	Polyether ether ketone
PET	Polyethylene terephthalate
PLA	Polylactide
PMMA	Polymethyl methacrylate
PP	Polypropylene
PS	Polystyrene
PQT	Poly quarter thiophene
PVA	Polyvinyl acetate
PVP	Polyvinylpyrrolidone
PZT	Piezo-electric
RFID	Radio-frequency identification
SE	Structural electronics
SEBS	Styrene-ethylene-butylene-styrene
SiO2	Silicon dioxide
SLA	Stereolithography
SLS	Selective laser sintering
SMT	Surface mount technology
SMD	Surface mount devices
SSAIL	Selective surface activation induced by a laser
TFT	Thin-film transistors
TPU	Thermoplastic polyurethane
UV	Ultraviolet
